# Exploring the brain in osteogenesis imperfecta: insights from the motor cortex and striatum of the *Brtl*^+/−^ murine model

**DOI:** 10.1186/s40478-026-02393-w

**Published:** 2026-07-30

**Authors:** Emma Lugli, Roberta Besio, Margherita Cavallo, Ludovica Gaiaschi, Wendy Pérez Franco, Gloria Milanesi, Antonella Forlino, Maria Grazia Bottone, Fabrizio De Luca

**Affiliations:** 1 Department of Biology and Biotechnology, University of Pavia, Pavia, Italy; 2 Biochemistry Unit, Department of Molecular Medicine, University of Pavia, Pavia, Italy

**Keywords:** Osteogenesis imperfecta, *Brtl* mouse model, Collagen, Motor cortex, Striatum, Oxidative stress

## Abstract

Osteogenesis imperfecta (OI) is a rare genetic disorder with heterogenous clinical severity, primarily caused to collagen I (COL1) defects. The unifying hallmark of OI pathophysiology is the presence of collagen I defects. Collagen I is one of the main components of the extracellular matrix (ECM) and in the central nervous system (CNS) defines brain architecture, supports the vascularisation and regulates crucial processes such as neuronal differentiation, migration, axonal growth and synaptogenesis. Previous findings highlighted the presence of cerebellar alterations in aged *Brtl* mice, a well-characterised murine model of dominant OI. The aim of the present work was to further investigate wheather a collagen I defect impacts CNS morphology and redox homeostasis. Morphology and redox potential were investigated in 18-month-old *Brtl*^+/−^ mice of both sexes, with a specific focus on the motor cortex and striatum, two regions that communicate with the cerebellum as part of the brain’s motor network. Through the application of bright-field microscopy, histological assessments were carried out using Nissl staining and Woelcke’s haematoxylin, while redox homeostasis was evaluated analysing the expression of several oxidative stress markers. Moreover, transmission electron microscopy was employed to further evaluate the impact of oxidative damage in presence of collagen I alterations on both neural and non-neural cell ultrastructure in the brains of *Brtl* mice. Overall, the cortical and striatal morphological alterations, the marked redox dysfunctions, as well as the ultrastructural changes, suggest that chronic oxidative stress, in presence of collagen I alteration, may result in morphological, ultrastructural and molecular modifications of the brain’s motor areas, paving the way for further research in this poorly investigated field.

**Graphical abstract:**

**Figure:**
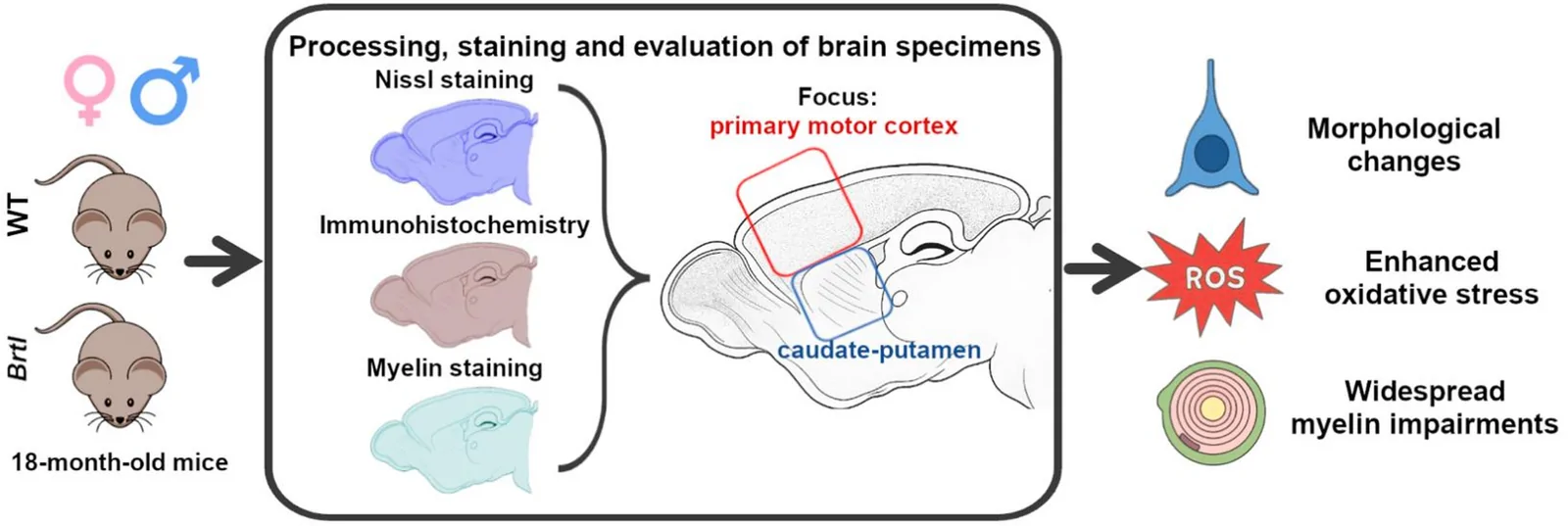

**Supplementary Information:**

The online version contains supplementary material available at https://doi.org/10.1186/s40478-026-02393-w.

## Introduction

With an incidence of 1:20.000 live births, Osteogenesis Imperfecta (OI) is a rare genetic disease yet representing the most common form of hereditary bone fragility [1]. OI is a connective tissue disorder with heterogounous clinical severity, primarily characterized by defects in collagen’s structure, folding, post-translational modification and processing [2]. Skeletal manifestations represent OI principal clinical hallmark and they include reduced bone density, increased risk of fractures, and progressive skeletal deformities; although the skeleton is the most prominently affected system, the disease shows a broader connective tissue involvement resulting in a wide spectrum of extraskeletal manifestations such as blue sclerae, hearing loss, abnormal odontogenesis, cardio-respiratory alterations as well as overall multi-systemic implications (e.g., widespread inflammation and immune system activation) [3–9].

Clinical classification encompasses several types with widely differing severity of both skeletal and extraskeletal manifestations, ranging from mild (Sillence Type I) to perinatally lethal (Type II), including moderate (Type IV) and severe, progressively deforming forms (Type III) [1, 10]. In the severe/moderately severe forms, extraskeletal symptoms also include delayed motor development and reduced locomotor abilities primarely linked to muscle abnormalities (reduced mass and functionality, altered fibre composition) and varying degrees of neurological dysfunctions: structural alterations including cerebellar displacement or hypoplasia and basilar impression, cerebrovascular defects (e.g., carotid–cavernous fistulas, cerebral aneurysms, and susceptibility to spontaneous intracranial or extracranial hemorrhage) as well as trigeminal neuralgia and sleep disturbances [11–18]. In addition, alterations of brain parenchyma, such as cerebral atrophy and communicating hydrocephalus, together with disturbances in neuronal migration and white matter organization, have also been reported (14, 19). Although OI neurological manifestations are largely viewed as secondary consequences of the underlying skeletal abnormalities, chronic pain and mobility limitations, increasing evidence suggest the disease has broader systemic implications [4–9].

Despite high clinical heterogeneity, most OI cases are attributed to collagen I (COL1) defects: more than 80% of all cases result from mutations in genes encoding the COL1 α chains (*Col1a1* and *Col1a2* genes), which lead to quantitative and/or qualitative protein alterations [2]. COL1 is a crucial constituent of the extracellular matrix (ECM), therefore defects in its synthesis or maturation can greatly compromise ECM homeostasis and physiological function in all COL1- containing tissues, including several components of the central nervous system (CNS) microenviroment [20]. Despite not being a major structural component in the brain, COL1 plays key roles in neuronal development, including axonal guidance and synaptogenesis, and its levels vary greatly depending on the developmental stage [20] and still serves structural functions in the adult brain, particularly in the meningeal and neurovascular compartment [21, 22]. In this regard, studies have highlighted how collagens, including COL1, are crucial components of meninges, both in the developing CNS and in the adult brain, and brain vasculature, where collagenous components are essential for the maintenance of the blood–brain-barrier [23–25].

Dysregulated ECM remodeling has been implicated in several neurological disorders, highlighting its role in modulating the neural microenvironment and brain pathology [26, 27]. Therefore, to address the potential implications of COL1 dysfunctions in the CNS, we employed the *Brtl*^+/−^ mouse, a murine model that well recapitulates the moderately severe human phenotype of dominant OI, carrying a heterozygous point mutation in the *Col1a1* gene responsible for α1(I)-G349C substitution [5, 28].

Beyond structural consequences, ECM dysfunction has also been associated with disturbances in neuronal and glial cells: an altered matrix composition can influence biochemical and mechanical cues to neurons and glial cells, thereby impairing synaptic stability, cell–cell signaling and glial responses that can exacerbate neural dysfunctions; transcriptomic analyses have revealed that dysregulation of extracellular matrix-associated genes is a hallmark feature across multiple neurological disorders [27, 29–32]. Particularly, ECM dysregulation and redox imbalance are closely interconnected in a bidirectional loop: while on one hand DNA damage and increased reactive oxygen species (ROS) may result in extracellular matrix alterations, ECM alterations, including COL1 abnormalities, can in turn lead to increased ROS and higher susceptibility to intracellular, such as mitochondrial oxidative stress reported in muscle cells and ultrastructural endoplasmic reticulum alterations [33–36]. This relationship appears particularly relevant in osteogenesis imperfecta, where changes in antioxidant and mitochondrial enzymes have been reported in murine models, indicating increased oxidative damage and integrated stress responses in diverse cell types: mutant collagen is partially retained inside the ER, causing ER ultrastructural alterations in fibroblasts as well as intracellular stress in osteoblasts; in addition, it is mutant collagen is partially retained inside the ER, causing ER ultrastructural alterations in fibroblasts as well as triggering intracellular stress reponses in osteoblasts; on the other hand, defective collagen is partially secreted into the ECM, altering its structure and interactions with other ECM components or cellular receptors [33, 37–41].

In this regard, the present study examined different brain areas of 18-month-old male and female mice to investigate whether alterations in COL1 may also have an impact on mice cerebrum. Recent preliminary findings in this model revealed alterations in the oxidative stress pathway in the cerebellum, a CNS region sharing both functional and pathophysiological connections with the motor cortex and striatum [37, 42, 43]. In particular, we focused on possible changes in two key brain regions: (i) the primary motor cortex, and (ii) the dorsal striatum, specifically, the caudate putamen in rodents. The dorsal striatum is anatomically and functionally connected to both the motor cortex and the cerebellum, forming what is known as the cortical-basal ganglia-cerebellar network. Within this network the motor cortex and striatum are part of the cortical-basal ganglia-thalamocortical loop. The cerebellum integrates into this system by forming direct connections with both the motor cortex and the striatum. Together, these neuroanatomical regions play a key role in the regulation of the motor functions: (i) the primary motor cortex is responsible for motor planning, control and execution; (ii) the dorsalstriatum participates in the modulation of movement; (iii) the cerebellum is key in coordination, balance, as well as motor refinement and learning [44]. These brain structures also share common vulnerability to oxidative damage, which may disrupt their integrated network, potentially leading to motor deficits [45–47]. Indeed, both granule cells in the cerebellum and pyramidal neurons in the motor cortex are highly metabolically active and sensitive to redox imbalances [48, 49]. Moreover, the striatum is also highly susceptible to mitochondrial dysfunctions and oxidative damage (in particular DNA and RNA oxidative damage), due to its high reliance on oxidative phosphorylation, and also shows a prominent production of ROS linked to dopamine metabolism [50, 51].

Considering the previously reported link between collagen defects and redox balance in OI, as well as the extensively studied role of the ECM in the brain, the aim of this work was to extend the analyses to other motor areas and investigate potential changes linked to COL1 alterations in aged *Brtl* mice, to better understand the long-term impact of OI pathophysiology on the CNS.

## Material and methods

### Mouse strain

The present study used CD1/129Sv/B6 *Col1a1*^+*/G349C*^ mice (*Brtl*), carrying a heterozygous G349C substitution in the α1(I) chain; and wildtype (WT) littermates, used as controls. Mutant and WT mice were maintained following standard experimental animal care protocols, in accordance with Italian Law, in the centralised animal facility of the University of Pavia (Italy). All experiments were approved by the OPBA (Body for the Protection of Animals) of the University of Pavia and by the Italian Ministry of Health (Protocol N 94/2024-PR), and were performed according to EU Directive 2010/63/EU.

Experiments were performed using 18-month-old mice (corresponding to advanced adulthood), focusing on the evaluation of long-term pathological alterations and potential differences in oxidative stress levels compared with a context characterized by chronic oxidative stress. 18-month-old Brtl mice were selected to evaluate the OI phenotype in late adulthood, corresponding to early elderly age in humans, as epidemiological evidence indicates that individuals with OI develop age-related comorbidities earlier than the general population, suggesting a progressive accumulation of disease-related alterations over time [52].

### Brain specimens’ preparation and experimental design

Before decapitation, 18-month-old male and female *Brtl* and wildtype (WT) mice were anaesthetised by isoflurane inhalation. Brains in their entirety were excised immediately after and then fixed by immersion in 4% paraformaldehyde (0.1 M phosphate buffer pH 7.4) for 24 h. Subsequently, tissues were dehydrated using an ascending graded ethanol scale followed by acetone and embedded in Paraplast X-TRA (Sigma Aldrich, Milan, Italy). Eight-micrometre-thick sagittal sections were serially cut using a rotary microtome and collected on silane-coated slides. Then, brain tissues were processed for morphological and histochemical evaluations using Nissl staining and Woelcke hematoxylin; additionally, immunohistochemical reactions were performed to assess the expression levels of specific oxidative stress markers i.e. SOD1, SOD2, GPx4, COX-IV, COX2, and iNOS. Referencing to standardized a mouse brain atlas (Allen Mouse Brain Atlas; https://mouse.brain-map.org/), specific regions of interest (i.e. CP and MOp) were subsequently selected for targeted analysis according to anatomical landmarks and cytoarchitectonic features; MOp layer V was identified and selected based on recognition of the characteristic morphpology of large pyramidal neurons [53–56]. Histochemical and immunohistochemical analyses were performed on n = 5 animals per group, while TEM investigations were carried out on one representative animal per group.

### Nissl staining

Nissl staining was performed to assess the degree of morphological alterations [57]. Brain sections were rehydrated and rinsed in distilled water; then, the sections were incubated in 1% cresyl violet (Sigma, St. Louis, MO, USA) solution for 3 min followed by differentiation in acetic acid in 100% ethanol (at 1:50,000 dilution) for 5 s. Lastly, sections were mounted using Eukitt (Kindler, Freiburg, Germany).

### Immunohistochemical reactions

Immunohistochemical assessments were performed as reported in previous publications and were carried out simultaneously on cerebrum slices from different experimental groups to avoid differences caused by procedural changes [58]. The sections were rehydrated and following the unmasking procedure (30 min in sodium citrate solution at 95 °C), immunohistochemical reactions were performed with commercial antibodies to investigate the expression, localization, and distribution of specific markers of oxidative stress pathway. After overnight incubation with the primary antibodies (Table 1), the slides were incubated for 1 h with the respective biotinylated secondary antibodies (Table 1) and an avidin biotinylated horseradish peroxidase complex (Vector Laboratories, Burlingame, CA, USA) was used to reveal the antigen/antibody interaction site. The 3,3′- diaminobenzidine (DAB) tetrahydrochloride peroxidase substrate (Sigma, St. Louis, MO, USA) was used as chromogen. Lastly, nuclear counterstaining was performed using Carazzi’s haematoxylin. Then, sections were dehydrated, cleared in xylene and mounted with Eukitt (Kindler, Freiburg, Germany).

**Table 1:** Primary and secondary antibodies with respective dilutions used for IHC reactions

	Antigen	Antibody	Dilution
Primary antibodies	Superoxide Dismutase-1 (SOD1)	GeneTex (Ivrine, CA, USA), Rabbit polyclonal IgG, Cat#GTX100554, RRID: AB_10618670	1:500
	Superoxide Dismutase-2 (SOD2)	Cell Signaling Technology (Danvers, MA, USA), Rabbit monoclonal IgG, Cat# 13,141, RRID: AB_2636921	1:200
	Glutathion Peroxidase 4 (GPx4)	Abcam (Cambridge, MA, USA), Rabbit polyclonal IgG, Cat# ab231174, RRID:AB_3073732	1:100
	Cytochrome C Oxidase Subunit IV (COX-IV)	Abcam (Cambridge, UK), Mouse monoclonal, Cat#AB33985, RRID: AB_879754	1:200
	Cyclooxygenase-2 (COX2)	Abcam (Cambridge, UK), Rabbit polyclonal, Cat#AB15191, RRID: AB_2085144	1:200
	Inducible Nitric Oxide Synthetase (iNOS)	GeneTex (Ivrine, CA, USA), Rabbit monoclonal, Cat#GTX636531, RRID: AB_2910003	1:50
	Collagen type I alpha 1 chain (COL1A1)	Santa Cruz Biotechnology (Dallas, TX, USA) Cat#sc-293182, Mouse monoclonal RRID: AB_2797597	1:100
Secondary antibodies	Biotinylated hoarse anti- mouse IgG	Vector Laboratories (Burlingame, CA, USA), Horse, Cat# PK-6102, RRID:AB_2336821	1:200
	Biotinylated hoarse anti- rabbit IgG	Vector Laboratories (Burlingame, CA, USA), Goat, Cat# PK-6101, RRID: AB_2336820	1:200

### Transmission electron microscopy evaluations

Transmission Electron Microscopy (TEM) evaluations were performed on one animal per experimental group, in order to obtain preliminary data on potential ultrastructural changes possibly associated with the investigated pathology; WT and *Brtl* brain specimens were processed for TEM analysis as previously reported [37, 59]. Briefly, the cerebrum (small samples of motor cortex and striatum of about 1 mm^3^) was fixed for 2 h by immersion in 1.5% glutaraldehyde (Polysciences, Inc., Warrington, PA, USA), buffered with 0.07 M cacodylate buffer (pH 7.4), containing 7% sucrose, followed by post-fixation in OsO_4_ (Sigma Chemical Co., St. Louis, MO, USA) in 0.1 M cacodylate buffer (pH 7.4) at 4 °C for 2 h, dehydrated in increasing concentrations of acetone and finally embedded in epoxy resin. Using a Reichert OM-U3 ultramicrotome, ultrathin sections (70–80 nm) were cut and collected on nickel grids, then stained with lead citrate. The sections were observed using a JEM 1200 EX II (JEOL, Peabody, MA, USA) electron microscope, equipped with a MegaView G2 CCD camera (Olympus OSIS, Tokyo, Japan) and operating at 100 kV.

Multiple sections (n = 3) and fields (n = 15) were examined for each sample to ensure consistency of the observed ultrastructural features. The analysis was intended to provide a preliminary qualitative evaluation of ultrastructural features.

### Woelcke’s haematoxylin staining

Staining with Woelcke’s hemtoxylin was performed to investigate potential myelin changes [60, 61]. Brain sections were rehydrated and rinsed in distilled water; then, the sections were incubated overnight in solution of ferric alum (5% ferric ammonium sulfate in distilled water). The following day the sections were quickly rinsed in distilled water and incubated for 2 h in Woelcke’s haematoxylin solution (alcoholic haematolxylin in distilled water and lithium carbonate solution); then, the sections were quickly differentiated in 80% ethanol, quickly dehydrated, cleared in xylene and mounted with Eukitt (Kindler, Freiburg, Germany). Lastly, stained specimens were evaluated using Leica DM6B WF bright-field microscope (Leica microsystems, Buccinasco, MI, Italy).

### Quantitative evaluations and statistical analyses

Stained specimens (i.e. 3 sections per animal at 48 µm intervals) were evaluated using a Leica DM6B WF bright-field microscope (Leica Microsystems, Buccinasco, MI, Italy) by a single blinded operator to avoid inter-rater variability and the images acquired with a Leica dfc 7000 t CCD camera; quantitative analyses were assessed on digitalized images of the brain sections using Image-J 1.48i software (NIH, Bethesda, MA, USA). For each histochemical and immunohistochemical analysis, quantitative evaluations were performed in 3 randomly chosen images/sections. The figures show the most representative images for each experimental condition. The following analyses were carried out on the different staining techniques: (i) for Nissl-stained sections, the density of shrunken neurons (number of shrunken cells/area in mm^2^) and cells (number of cells/area in mm^2^) were performed in the motor cortex and caudate-putamen, respectively; the tissue area was kept constant to ensure consistency across all analyses (ii) for immunolabelled sections, the extent of the immunopositive signal was measured as the mean of intensity value over the area and indicated as optical density (OD), with at least 10 measurements of OD performed per image: [62, 63]; immunopositive cell density (CD) (number of immunopositive cells/area in mm^2^) were also measured; (iii) sections stained with Woelcke’s haematoxylin were used to carry out quantitative analyses of myelin staining, to evaluate possible changes in the expression levels of myelin: a score from 0 (no staining) to 5 (intense, uniform staining) has been defined to identify different levels of staining intensity (as exemplified in Fig. S1). For OD analyses, images were acquired with exposure settings preventing pixel saturation and inverted to convert DAB signal into positive signal correlating with optical density; polygon selection tool was used to define regions of interest based on spatial distribution and cell types. Labeling intensity was measured as mean intensity over the selected area and background subtraction was performed using unstained regions from the same sections.

The results are expressed as mean ± SEM. The D’Agostino & Pearson, Anderson–Darling, Kolmogorov–Smirnov and Shapiro–Wilk tests were performed to evaluate the normality distributions of data. Subsequently, collected results were processed to verify statistically significant differences. Male and female mice were analyzed separately; within each sex, group comparisons were performed analysed using the Mann-Witney test for non-normally distributed data, while for normally distributed data the analysis was performed with an Unpaired t-test. Differences were considered statistically significant for *p* < 0.05 (*), *p* < 0.01 (**) and *p* < 0.001 (***).

## Results

**Fig. 1:**
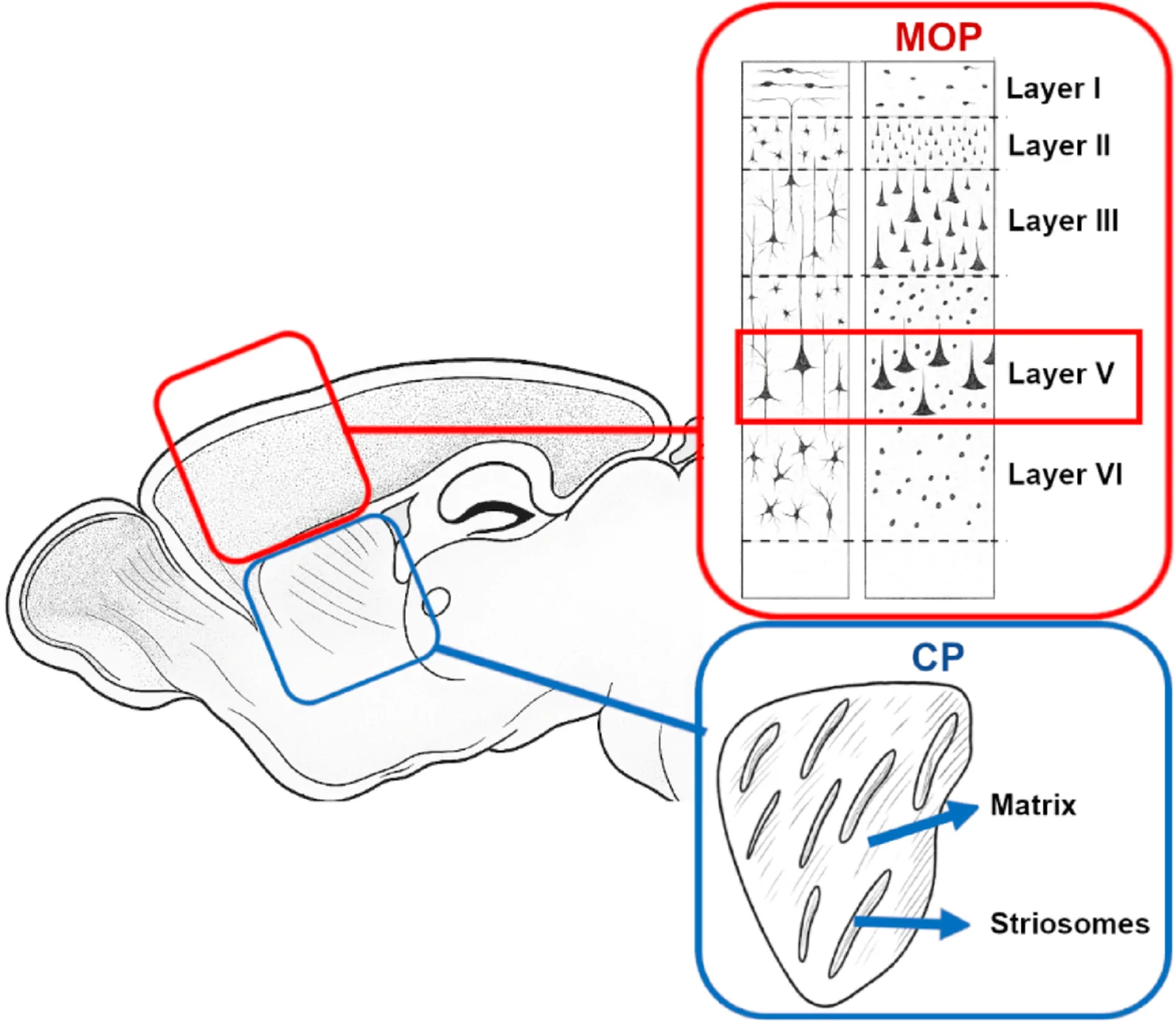
Schematic illustration of a mouse sagittal brain highlighting the CNS areas investigated in this study: the primary motor cortex (MOp), with particular attention to pyramidal layer V, and the striatum caudate-putamen (CP)

To investigate the potential impact of COL1 mutations in the brain of *Brtl* animals, which showed a reduced COL1A1 immunopositivity in the meninges and blood vessels (Fig. S2; Table S1), we employed complementary approaches combining (i) histochemical, (ii) immunohistochemical, (iii) and TEM assessments. In light of the previously reported cerebellar alterations, to further investigate the CNS motor areas, this work was focused on the possible alterations/modifications of the primary motor cortex (pyramidal layer V) and the striatum (caudate-putamen) (Fig. 1).

### Morphological changes of the motor cortex and striatal caudate-putamen

Nissl stain, often used to highlight nucleic acids and aggregates of rRNAs and endoplasmic reticulum, known as Nissl bodies, typical of neuronal cell types [64, 65], was employed to evaluate neuronal structure. Specifically, Nissl stain can highlight shrunken-hyperchromatic cells, intensely-stained neurons with a condensed and elongated abnormal morphology (often characterised by pyknotic nuclei): these changes in neuronal morphology are generally associated with cytological alterations including dysfunctions of mitochondria and endoplasmic reticulum [66]. In the present study, shrunken cells density was evaluated in the inner pyramidal layer of the motor cortex, while in the caudate-putamen we performed only cell density analyses due to the poor discrimination between neuronal populations within the striatum, making it challenging to reliably identify shrunken neurons.

**Fig. 2:**
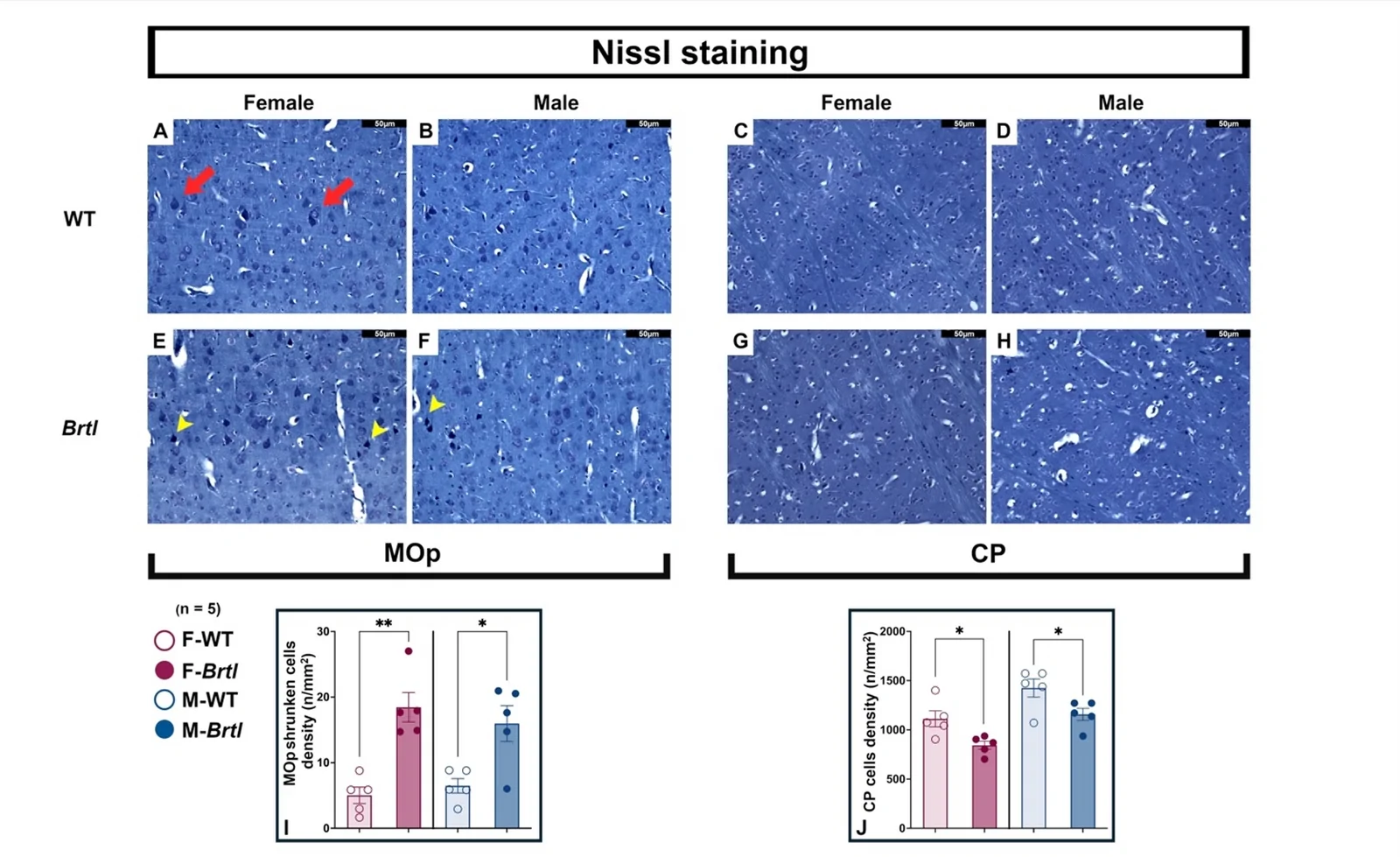
Representative micrographs and corresponding quantitative analysed of Nissl histochemical staining in sagittal section of the primary motor cortex (MOp) and caudate-putamen (CP), from male (**B**, **D**, **F**, **H**) and female (**A**, **C**, **E**, **G**) wildtype (WT; **A**–**D**) and Brtl mice (**E**–**H**). Magnification: 10 × (**C**, **D**, **G**, **H**) and 20 × (**A**, **B**, **E**, **F**). Bar graphs: density of shrunken pyramidal neuron in the Mop (**I**) and cell density count in the CP (**J**). Red arrows exemplify large pyramidal neurons of MOp layer V; yellow arrowheads indicate hyperchromatic pyramidal neurons, more intensely stained and with a shrunken, collapsed morphology. *p*-values: (*) *p* < 0.05, and (**) *p* < 0.01

Figure 2 shows Nissl-stained sections of the sagittal primary motor cortex (MOp) and striatum caudate-putamen (CP). Subpanels A–D and E–H correspond to WT and Brtl mice, respectively. Within each genotype, subpanels A, C, E, and G represent females, whereas subpanels B, D, F, and H represent males. Nissl staining in the inner pyramidal layer (layer V) of the motor cortex revealed intensely stained large pyramidal neurons (red arrows in Fig. 2A). Pyramidal neurons show a well-defined morphology: large soma, intensely stained nucleolus, and lightly stained cytoplasm with evident Nissl substance; the prominent nucleolus is strongly stained thanks to the presence of RNAs involved in ribosomal assembly, while Nissl bodies account for the high amount of RNAs in either free ribosomes or endoplasmic reticulum-associated ribosomes. Additionally, we can clearly observe the intensely stained apical dendritic trunk (proximal region). Few smaller neurons as well as glial cells can also be observed; the latter, can be recognised from their intensely stained rounded nucleus paired with a lighter cytoplasm that forms a sort of ‘halo’. Moreover, qualitative investigations on Nissl-stained sections of motor cortex layer V revealed sparse hyperchromatic pyramidal neurons, more intensely stained and with a shrunken, collapsed morphology, which appear more frequent in male and female *Brtl* animals compared to control animals (Fig. 2). Quantitative results evidenced an overall significantly higher density of shrunken neurons in the motor cortex layer V in both male and female *Brtl* mice when compared to the respective controls (F-WT vs F-*Brtl* *p* < 0.001; M-WT vs M-*Brtl* *p* = 0.01) (Fig. 2I; Table S1).

Similarly to the cortex, Nissl staining highlights the neuronal population of the striatum; neurons display a well-delineated cytoplasm and an intensely stained nucleolus. Compared to the pyramidal neurons of layer V in the motor cortex, striatal neurons of the caudate-putamen appear smaller and rounder which does not allow clear distinction between different neuronal populations. Overall, the striatum shows a homogenous organisation, lacking the laminar structure typical of other brain regions including the cortex. We also observe sparse hyperchromatic cells with shrunken morphology and pyknotic nuclei, potentially shrunken striatal neurons. Quantitative analyses regarding striatal cell density revealed a significant reduction in the caudate-putamen region of *Brtl* mice, compared to the respective controls (F-WT vs F-*Brtl* *p* = 0.02; M-WT vs M-*Brtl* *p* = 0.0425) (Fig. 2J; Table S3).

### Immunohistochemical analyses of specific oxidative stress markers in the motor cortex and striatal caudate-putamen

Building on previous evidence regarding redox alteration in the cerebellum of *Brtl* animals and on the changes revealed by morphological assessments with Nissl staining in the motor cortex and caudate-putamen of mutant animals, we carried out immunohistochemical analyses targeting these CNS regions to further investigate the redox equilibrium in brain areas responsible for motor control, using specific oxidative stress markers (i.e. SOD1, SOD2, GPx4, COX-IV, COX2, and iNOS). The specificity of immunostaining in neurons or glial cells is supported by consistent staining patterns across independent biological samples and the absence of signal in these cell types in no-primary-antibody controls (Figs. S3–S8); IgG isotype controls were not included.

**Fig. 3:**
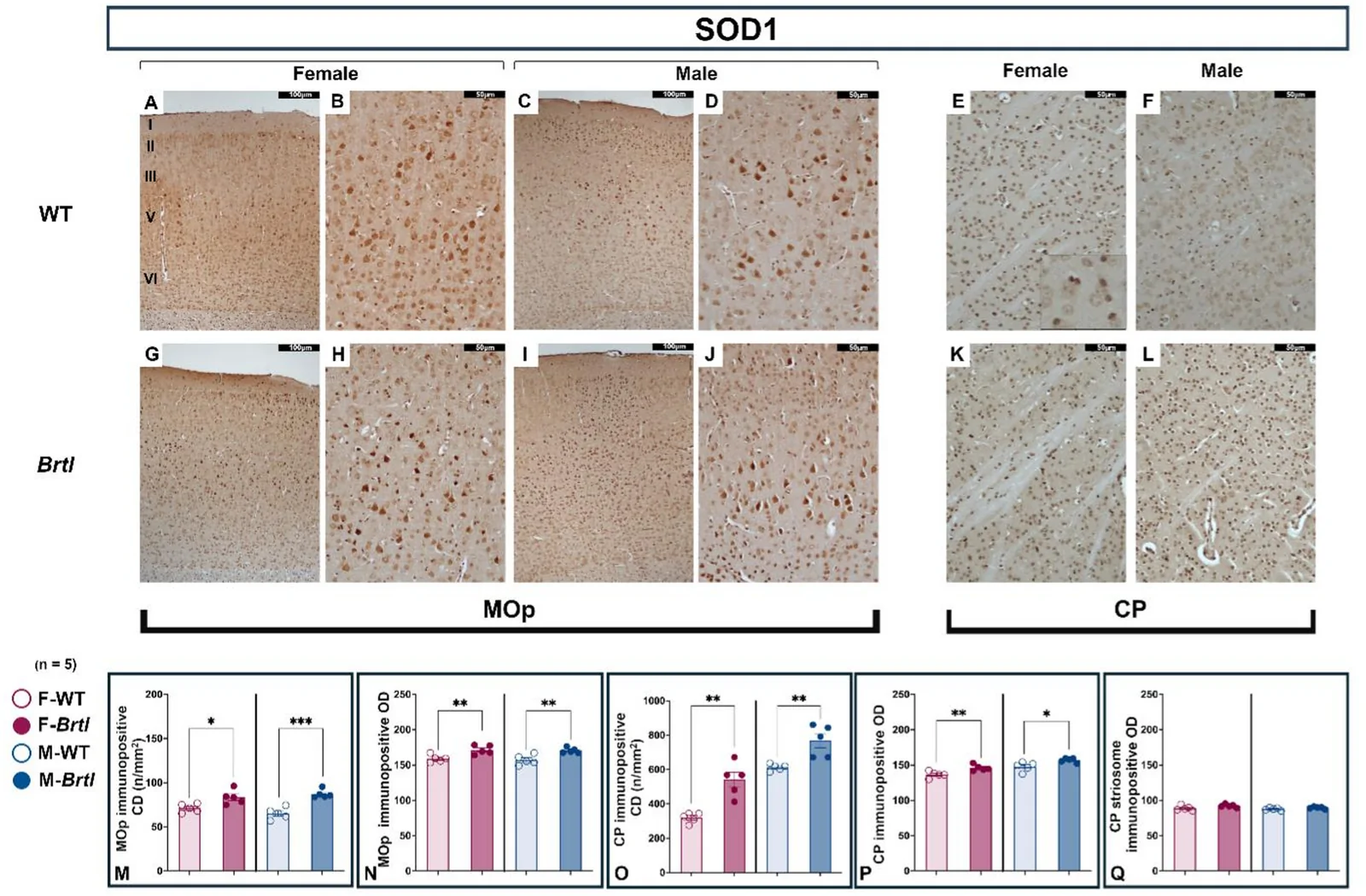
Representative micrographs and corresponding quantitative analysed of SOD1 immunohistochemical reaction in sagittal section of the primary motor cortex (MOp) and caudate-putamen (CP), from wildtype (WT; **A**–**F**) and Brtl mice (**G**–**L**); insert in E (WT female) exemplifies SOD1-immunopositive signal in neurons and glial cells of the CP. Roman numerals in panel **A** indicate MOp layers. Magnification: 10 × (**A**, **C**, **G**, and **I**) and 20 × (**B**, **D**, **E**, **F**, **H**, **J**, **K**, and **L**); **B**, **D**, **H**, and **J** are magnified views of **A**, **C**, **G**, and **I**, respectively. Bar graphs: SOD1-immunopositive cell density (CD) and optical density (OD) in panels M-Q. *p*-values: (*) *p* < 0.05, (**) *p* < 0.01, and (***) *p* < 0.001

Starting with the superoxide dismutases, crucial antioxidant enzymes, the evaluation of SOD1 (Fig. 3) revealed significantly elevated levels in *Brtl* mice primary motor cortex and striatum, evidenced by an increase in the density of immunopositive cells and in optical density (OD). Subpanels A–F and G–L correspond to WT and Brtl mice, respectively. Within each genotype, subpanels A, B, G, H, E and K represent females, whereas subpanels C, D, I, J, F and L represent males.

In the motor cortex, widespread SOD1 immunostaining can be observed in all cortical layers both in *Brtl* and control groups, showing a strong staining localised in the whole cellular soma, particularly in the cytoplasm and nucleolus; some neurons also show slight SOD1 immunopositivity localised at the level of the dendrites. Several large intensely stained neurons can be observed in the inner pyramidal layers of the motor cortex (Fig. 3B, D, H and J). Focusing on the innermost cortical layers, quantitative analyses revealed a substantial increase in the number of SOD1 immunopositive neurons in the motor cortex layer V of *Brtl* groups (F-WT vs F-*Brtl* *p* = 0.02; M-WT vs M-*Brtl* *p* < 0.001) (Fig. 3M; Table S4). Additionally, optical density quantitative analyses of the cortical pyramidal neurons also revealed a significant increase of SOD1 immunopositive OD in both male and female *Brtl* groups (F-WT vs F-*Brtl* *p* = 0.010; M-WT vs M-*Brtl* *p* = 0.006) (Fig. 3N; Table S5).

In the caudate-putamen, SOD1-immunopositive cells can be observed both in the striosomes and matrix, where neurons and glial cells show a marked SOD1 immunolabeling (Fig. 3E, insert). Specifically, both male and female *Brtl* animals show a stronger SOD1-immunopositivity, particularly evident for female mutant animals compared to the respective controls. In line with the observations made in the motor cortex, the quantitative analyses in the caudate-putamen region evidenced that *Brtl* mice of both sexes display a significant increase in the density of SOD1-immunopositive cells (F-WT vs F-*Brtl* *p* = 0.001; M-WT vs M-*Brtl* *p* = 0.006) (Fig. 3O; Table S4). The optical density analyses of individual immunopositive neurons revealed a significant increase in SOD1-immunopositive OD in mutant animals compared to the controls (F-WT vs F-*Brtl* *p* = 0.010; M-WT vs M-*Brtl* *p* = 0.02); conversely, no changes were displayed in striosomal OD (F-WT vs F-*Brtl* *p* = 0.1097; M-WT vs M-*Brtl* *p* = 0.1388) (Fig. 3P and Q, respectively; Table S5).

**Fig. 4:**
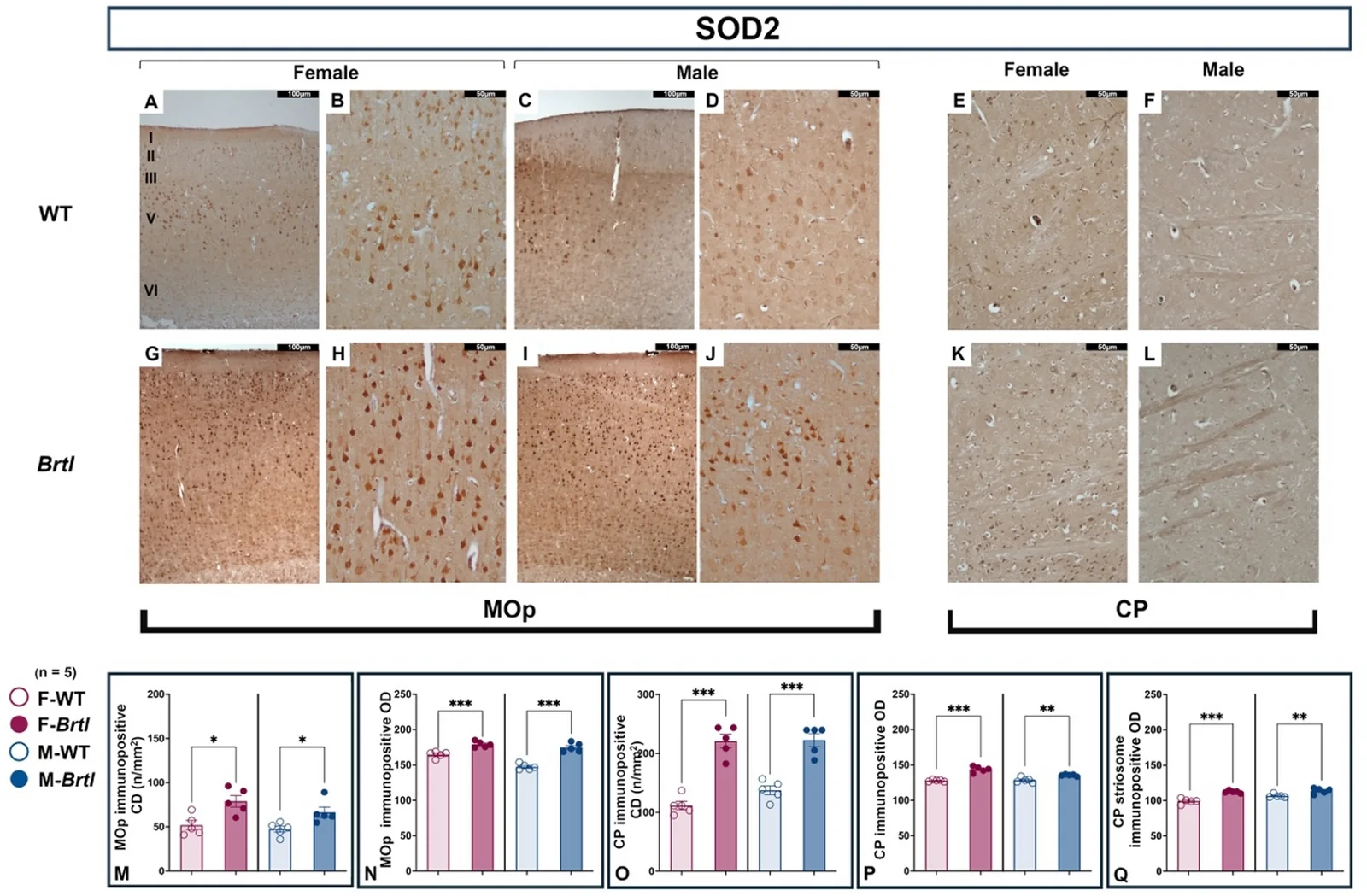
Representative micrographs and corresponding quantitative analysed of SOD2 immunohistochemical reaction in sagittal section of the primary motor cortex (MOp) and caudate-putamen (CP), from wildtype (WT; **A**–**F**) and Brtl mice (**G**–**L**). Roman numerals in panel **A** indicate MOp layers. Magnification: 10 × (**A**, **C**, **G**, and **I**) and 20 × (**B**, **D**, **E**, **F**, **H**, **J**, **K**, and **L**); **B**, **D**, **H**, and **J** are magnified views of **A**, **C**, **G**, and **I**, respectively. Bar graphs: SOD2-immunopositive cell density (CD) and optical density (OD) in panels M-Q. *p*-values: (*) *p* < 0.05, (**) *p* < 0.01, and (***) *p* < 0.001

Similarly to SOD1, SOD2 evaluation indicated increased levels in *Brtl* samples; yet, SOD2 immunohistochemical reactions in the motor cortex revealed a much different distribution between *Brtl* and control groups (Fig. 4). Subpanels A–F and G–L correspond to WT and Brtl mice, respectively. Within each genotype, subpanels A, B, G, H, E and K represent females, whereas subpanels C, D, I, J, F and L represent males. Control animals display a more scattered distribution of SOD2-immunopositive cells, particularly localised in the pyramidal layers; conversely, *Brtl* mice display a more widely distributed SOD2 immunopositive cells, which localises in all cortical layers. In the pyramidal layer, SOD2 immunostaining can be observed in the whole soma of cells morphologically resembling pyramidal neurons and also extends to the dendrites, which appear highly stained specifically in male and female mutant animals (Fig. 4B, D, H, and J). Consistently, quantitative analyses showed that *Brtl* mice of both sexes have a significantly higher density of SOD2-immunopositive cells in layer V of the motor cortex (F-WT vs F-*Brtl* *p* = 0.01; M-WT vs M-*Brtl* *p* = 0.0218). Additionally, the results of the optical density analyses indicated that SOD2-immunopositive OD in layer V pyramidal neurons is significantly higher in mutant animals: particularly, both male and female *Brtl* groups showed an extremely significant increase compared to the respective controls (F-WT vs F-*Brtl* *p* < 0.001; M-WT vs M-*Brtl* *p* < 0.001) (Fig. 4; Tables S6 and S7).

SOD2-immunostaining in the matrix and striosomes of the caudate-putamen region localises primarily at the cellular cytoplasmic level (Fig. 4). In contrast to the motor cortex, several SOD2-immunonegative cells can be observed in the striatal matrix and the signal appears overall less intense. In the striatum, quantitative analyses of SOD2-immunopositive cell density revealed a significant increase in both *Brtl* groups (F-WT vs F-*Brtl* *p* < 0.001; M-WT vs M-*Brtl* *p* < 0.001). Additionally, the analyses demonstrated also a significant increase in SOD2 optical density in individual immunopositive cells (F-WT vs F-*Brtl* *p* < 0.001; M-WT vs M-*Brtl* *p* = 0.003) as well as in signal specifically localised to striatal patches (F-WT vs F-*Brtl* *p* < 0.001; M-WT vs M-*Brtl* *p* = 0.009) (Fig. 4; Tables S6 and S7).

**Fig. 5:**
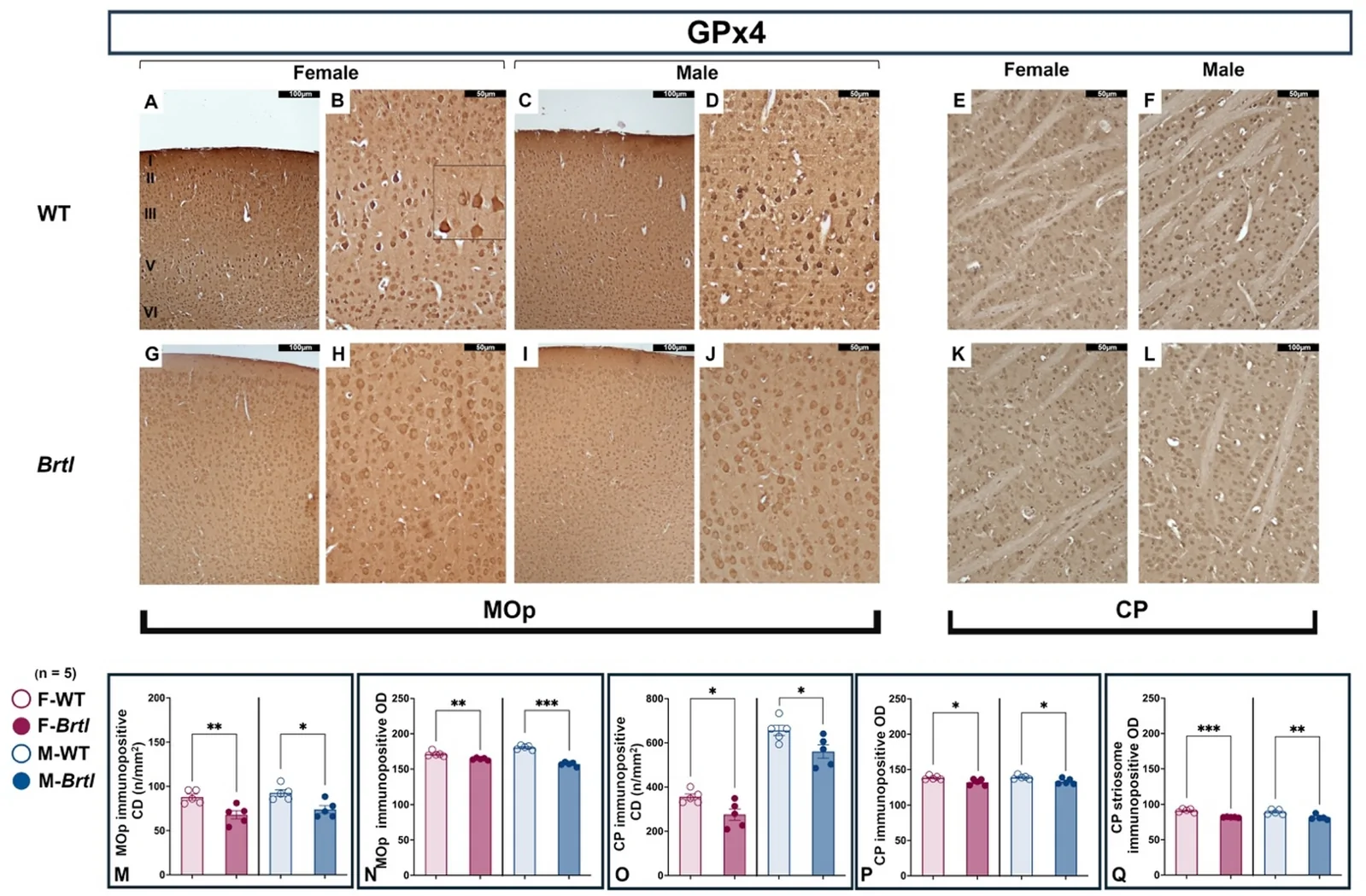
Representative micrographs and corresponding quantitative analysed of GPx4 immunohistochemical reaction in sagittal section of the primary motor cortex (MOp) and caudate-putamen (CP), from wildtype (WT; **A**–**F**) and Brtl mice (**G**–**L**); the insert in panel **B** (WT female) highlights strong GPx4 cytoplasmic immunostaining extending to the proximal region of apical dendrites, in the large pyramidal neurons of the motor cortex-layer V. Roman numerals in panel **A** indicate MOp layers. Magnification: 10 × (**A**, **C**, **G**, and **I**) and 20 × (**B**, **D**, **E**, **F**, **H**, **J**, **K**, and **L**) (insert in **B** 40 ×); **B**, **D**, **H**, and **J** are magnified views of **A**, **C**, **G**, and **I**, respectively. Bar graphs: GPx4-immunopositive cell density (CD) and optical density (OD) in panels M-Q. *p*-values: (*) *p* < 0.05, (**) *p* < 0.01, and (***) *p* < 0.001

In contrast to superoxide dismutases, glutathione peroxidase 4 (GPx4) levels exhibited a decrease in *Brtl* mice. Immunostained sections show a marked GPx4 cytoplasmic staining in layer V pyramidal neurons of control mice, which appears much lighter in both *Brtl* groups (Fig. 5). Moreover, in some neurons the staining also extends to the proximal portion of the apical dendrites, particularly evident in wildtype mice (Fig. 5B, insert). Subpanels A–F and G–L correspond to WT and Brtl mice, respectively. Within each genotype, subpanels A, B, G, H, E and K represent females, whereas subpanels C, D, I, J, F and L represent males.

Furthermore, the quantitative analyses revealed significant changes in cortical GPx4 immunostaining between control and *Brtl* mice. Our data evidenced a reduction in both the density of GPx4 immunopositive cells (F-WT vs F-*Brtl* *p* = 0.008; M-WT vs M-*Brtl* *p* = 0.01). Additionally, mutant animals of both sexes also showed a significant reduction of GPx4 OD in layer V of the motor cortex, even though more marked in male groups (F-WT vs F-*Brtl* *p* = 0.0042; M-WT vs M-*Brtl* *p* < 0.001) (Fig. 5; Tables S8 and S9).

Cytoplasmic GPx4-immunostaining in the dorsal striatum appears overall sparser compared to the cortex (Fig. 5). Nevertheless, upon quantification, GPx4-immunopositive cell density (F-WT vs F-*Brtl* *p* = 0.03; M-WT vs M-*Brtl* *p* = 0.04) and individual cells OD analyses revealed a significant decrease of GPx4 levels in *Brtl* mice (F-WT vs F-*Brtl* *p* = 0.03; M-WT vs M-*Brtl* *p* = 0.03); moreover, GPx4 mean OD in the striosomal regions appears significantly decreased in both male and female mutant animals compared to the respective control groups(F-WT vs F-*Brtl* *p* < 0.001; M-WT vs M-*Brtl* *p* = 0.009) (Fig. 5; Tables S8 and S9).

**Fig. 6:**
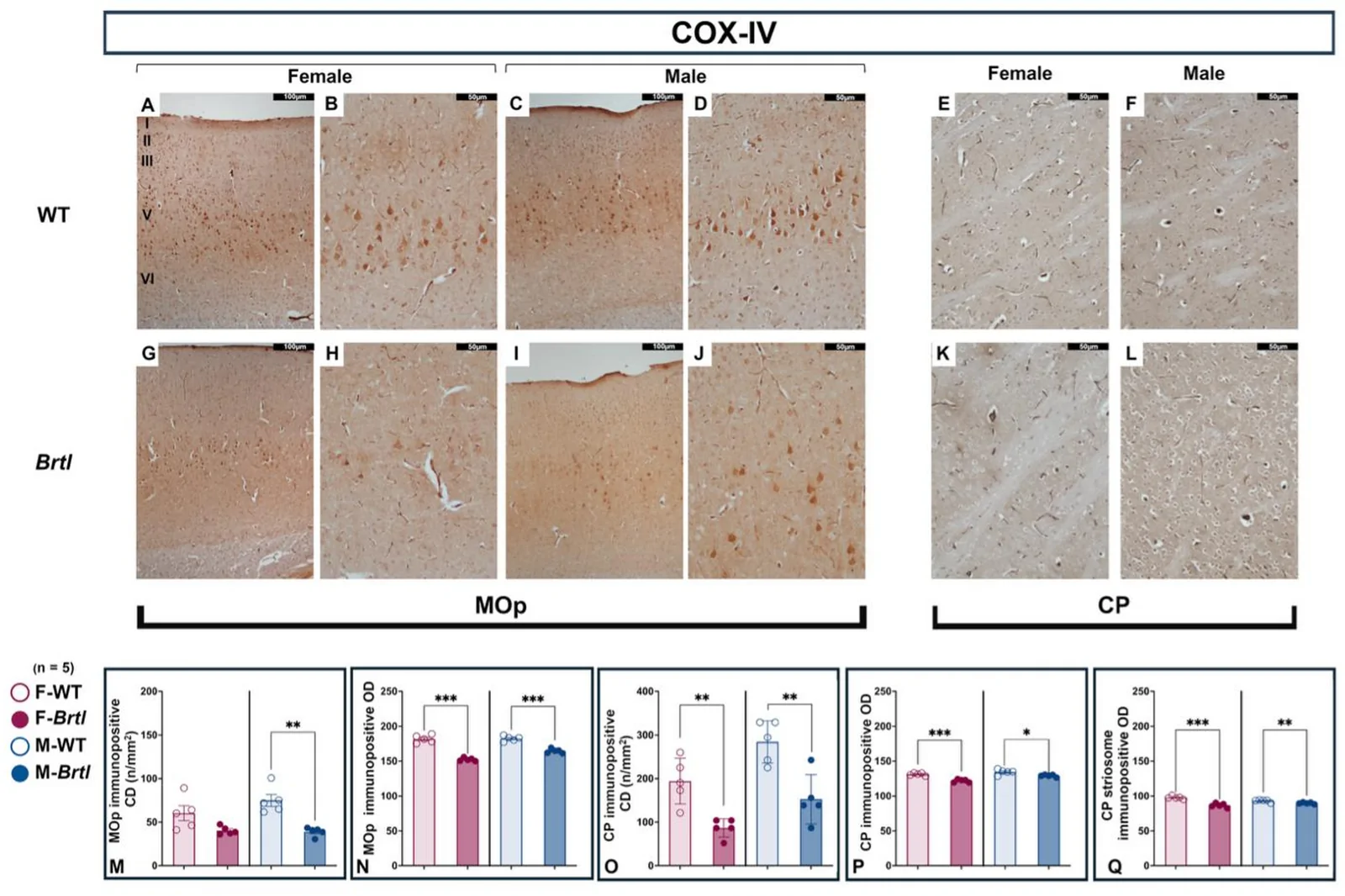
Representative micrographs and corresponding quantitative analysed of COX-IV immunohistochemical reaction in sagittal section of the primary motor cortex (MOp) and caudate-putamen (CP), from wildtype (WT; **A**–**F**) and Brtl mice (**G**–**L**). Roman numerals in panel **A** indicate MOp layers. Magnification: 10 × (**A**, **C**, **G**, and **I**) and 20 × (**B**, **D**, **E**, **F**, **H**, **J**, **K**, and **L**); **B**, **D**, **H**, and **J** are magnified views of **A**, **C**, **G**, and **I**, respectively. Bar graphs: COX-IV immunopositive cell density (CD) and optical density (OD) in panels M-P. *p*-values: (*) *p* < 0.05, (**) *p* < 0.01, and (***) *p* < 0.001

COX-IV, a mitochondrial respiratory chain marker (complex IV), was also significantly reduced in *Brtl* vs. control animals. In the primary motor cortex, COX-IV immunostaining primarily localises in the pyramidal layers; focusing on the innermost pyramidal layer, we observe a strong cytoplasmic staining in the soma of layer V neurons, visibly more prominent in control animals compared to the *Brtl* groups (Fig. 6). Subpanels A–F and G–L correspond to WT and Brtl mice, respectively. Within each genotype, subpanels A, B, G, H, E and K represent females, whereas subpanels C, D, I, J, F and L represent males.

The results of the quantitative analyses revealed a substantial reduction of COX-IV immunostaining in *Brtl* male and female mice. Male *Brtl* groups displayed a significant reduction in the density of immunopositive cells in the innermost pyramidal layer of the motor cortex; *Brtl* female showed a lower density of immunopositive neurons as well, despite being non-significant (F-WT vs F-*Brtl* *p* = 0.05; M-WT vs M-*Brtl* *p* < 0.001). Furthermore, an extremely significant reduction of COX-IV optical density was detected in both *Brtl* groups (F-WT vs F-*Brtl* *p* < 0.001; M-WT vs M-*Brtl* *p* < 0.001) (Fig. 6; Tables S10 and S11).

In the dorsal striatum, COX-IV immunopositive signal localises within the cytoplasm, primarily in the cell types localised in the matrix, yet showing a less intense signal compared to cortical pyramidal neurons (Fig. 6). Quantitative analyses of the caudate-putamen indicate a marked reduction of COX-IV immunopositive cell density and optical density in *Brtl* animals. Indeed, both male and female mutant mice displayed a very significant decrease in immunopositive CD (F-WT vs F-*Brtl* *p* = 0.003; M-WT vs M-*Brtl* *p* = 0.004); additionally, male and female *Brtl* groups showed a significant and extremely significant reduction in COX-IV immunopositive OD, respectively (F-WT vs F-*Brtl* *p* < 0.001; M-WT vs M-*Brtl* *p* = 0.02). Lastly, we detected a very significant increase of striosomal COX-IV immunopositive OD in both *Brtl* genders (F-WT vs F-*Brtl* *p* < 0.001; M-WT vs M-*Brtl* *p* = 0.008) (Fig. 6; Tables S10 and S11).

**Fig. 7:**
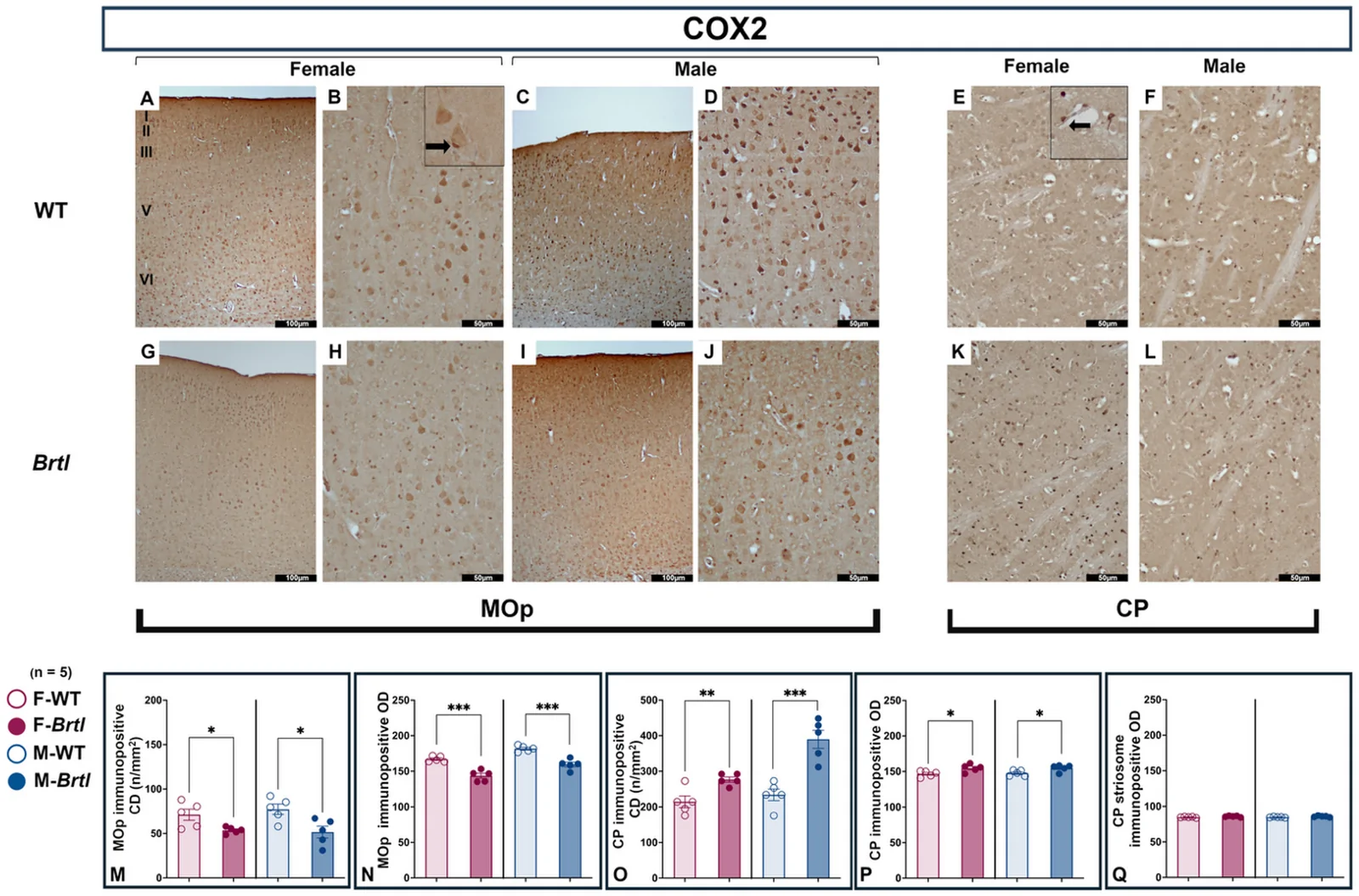
Representative micrographs and corresponding quantitative analysed of COX2 immunohistochemical reaction in sagittal section of the primary motor cortex (MOp) and caudate-putamen (CP), from wildtype (WT; **A**–**F**) and Brtl mice (**G**–**L**); inserts in panels **B** and **E** (WT female) exemplify COX2 immunopositive signal in smaller cell types, likely astrocytes, enveloping a neuron and envrapping a blood vessel, respectively. Roman numerals in panel **A** indicate MOp layers. Magnification: 10 × (**A**, **C**, **G**, and **I**) and 20 × (**B**, **D**, **E**, **F**, **H**, **J**, **K**, and **L**); **B**, **D**, **H**, and **J** are magnified views of **A**, **C**, **G**, and **I**, respectively. Bar graphs: COX2-immunopositive cell density (CD) and optical density (OD) in panels M-Q. *p*-values: (*) *p* < 0.05, (**) *p* < 0.01, and (***) *p* < 0.001

To further investigate the contribution of redox imbalances on cortical and striatal homeostasis, we also performed an immunohistochemical reaction against cycloxigenase 2 (COX2) (Fig. 7). Overall, COX2, often associated with inflammation and oxidative stress, was reduced in cortical neurons in *Brtl* mice (both cell density and intensity), but in the striatum there was elevated COX2 in glial/non‐neuronal cells. This pattern hints at a shift from neuronal oxidative/inflammatory activity in cortex toward glial inflammatory responses in the striatum, although this hypothesis remains to be tested emplying specific inflammatory markers.

In the motor cortex, the staining revealed a widespread COX2-immunopositivity for this enzyme in the soma of layer V neurons, which appear overall more intensely stained in male groups than in females; additionally, sparse neurons display a light COX2-immunostaining localised also in the most proximal region of the apical dendrites (Fig. 7). Subpanels A–F and G–L correspond to WT and Brtl mice, respectively. Within each genotype, subpanels A, B, G, H, E and K represent females, whereas subpanels C, D, I, J, F and L represent males.

Numerous smaller cell types with a very intense nuclear staining localised both in the cytoplasm and, less frequently, in their processes likely represent glial cells, particularly microglial cells and astrocytes, where the latter can be observed enwrapping blood vessels or in close proximity to large pyramidal neurons enveloping their somata (Fig. 7B, insert). Quantitative analyses revealed substantial alterations in the density of COX2-immunopoitivity between mutant and control animals. Indeed, male and female *Brtl* groups showed a significant decrease in COX2-immunopositive cell density (F-WT vs F-*Brtl* *p* = 0.03; M-WT vs M-*Brtl* *p* = 0.02); moreover, COX2 optical density analyses evidenced an extremely significant reduction in *Brtl* mice of both sexes (F-WT vs F-*Brtl* *p* < 0.001; M-WT vs M-*Brtl* *p* < 0.001) (Fig. 7; Tables S12 and S13).

Differently from what observed in the cortex, striatal neurons display a much sparser and lower COX2-immunopositive signal; conversely, the glial component appears intensely stained: smaller non-neuronal cells, likely microglial cells, can be observed while bigger cell types, presumably astrocytes, can be identified as they enwrap blood vessels (Fig. 7E, insert). The quantitative analyses for striatal COX2-immunostaining on the non-neuronal component indicated a significant increase of COX2-immunopositive cell density (F-WT vs F-*Brtl* *p* = 0.008; M-WT vs M-*Brtl* *p* < 0.001) and OD in the caudate-putamen of *Brtl* groups (F-WT vs F-*Brtl* *p* = 0.03; M-WT vs M-*Brtl* *p* = 0.03). Conversely, in striosomal patches we detected no differences in COX2-immunopositive OD (F-WT vs F-*Brtl* *p* = 0.10; M-WT vs M-*Brtl* *p* = 0.10) (Fig. 7; Tables S12 and S13).

iNOS, the inducible form of nitrogen oxide synthase, was also differentially regulated between wildtype and mutated animals: in the cortex of *Brtl* mice there was a marked reduction in iNOS immunopositivity (both in number of positive neurons/cells and in OD), more pronounced in males; in the striatum, reductions were sex‐specific (significant in male mutants, not in females). Together, these data suggest that nitric oxide-mediated oxidative or nitrosative stress pathways are downregulated in neurons of *Brtl* mice, or that their activation is impaired, possibly contributing to altered redox homeostasis.

**Fig. 8:**
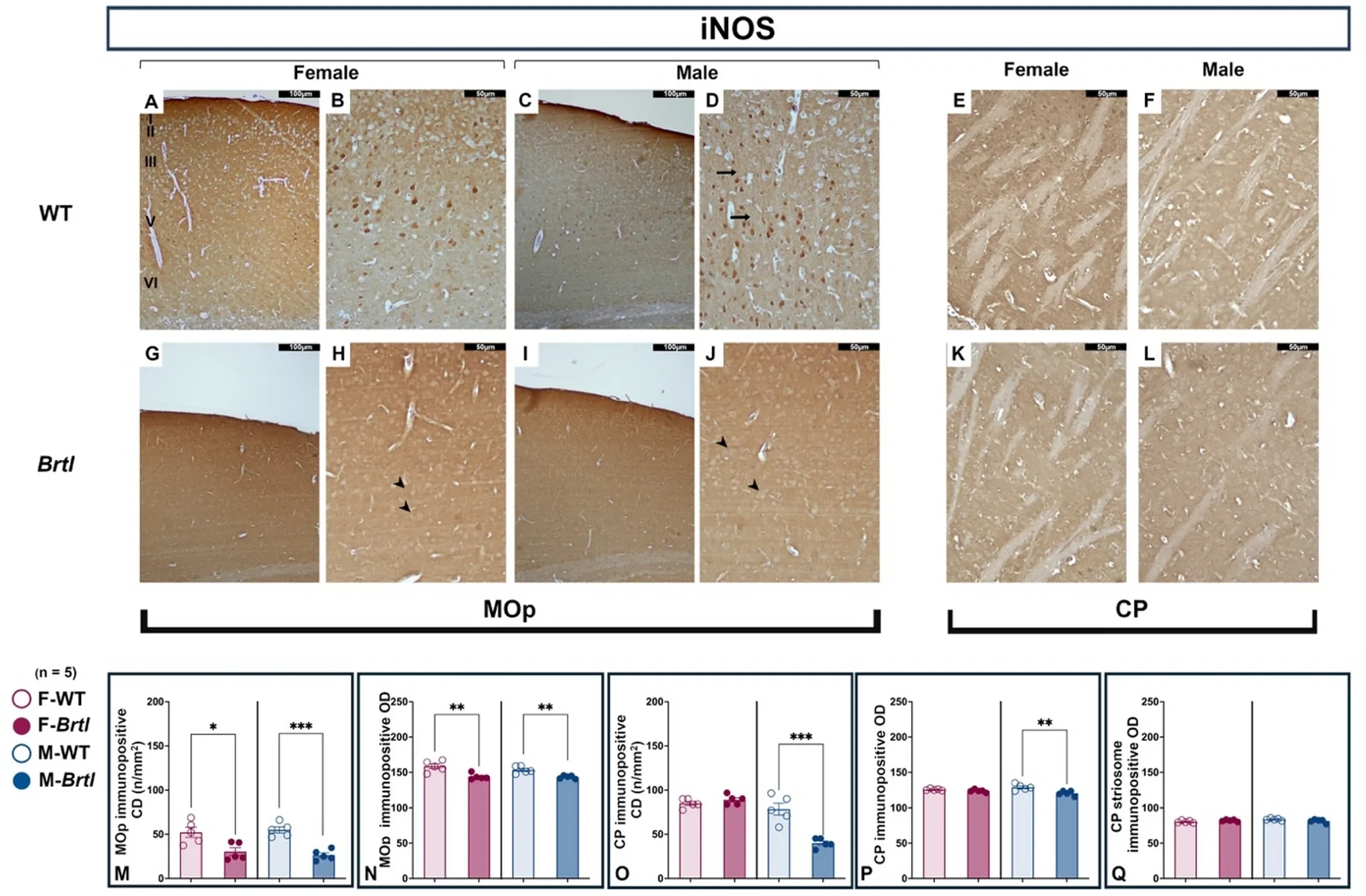
Representative micrographs and corresponding quantitative analysed of iNOS immunohistochemical reaction in sagittal section of the primary motor cortex (MOp) and caudate-putamen (CP), from wildtype (WT; **A**–**F**) and Brtl mice (**G**–**L**); black arrows in panel **D** (WT male) exemplify iNOS-immunopositive signal localised in the dendrites of MOp large puyramidal neurons; black arrowheads in panels **H** and **J** point to iNOS-immunonegative MOp pyramidal neurons. Roman numerals in panel A indicate MOp layers. Magnification: 10 × (**A**, **C**, **G**, and **I**) and 20 × (**B**, **D**, **E**, **F**, **H**, **J**, **K**, and **L**); **B**, **D**, **H**, and **J** are magnified views of **A**, **C**, **G**, and **I**, respectively. Bar graphs: iNOS-immunopositive cell density (CD) and optical density (OD) in panels M-Q. *p*-values: (*) *p* < 0.05, (**) *p* < 0.01, and (***) *p* < 0.001

Immunohistochemical reaction revealed a strong iNOS-immunopositivity in the cortical innermost layers of male and female wildtype animals, and a considerably less prominent staining in *Brtl* mice (Fig. 8). Subpanels A–F and G–L correspond to WT and Brtl mice, respectively. Within each genotype, subpanels A, B, G, H, E and K represent females, whereas subpanels C, D, I, J, F and L represent males. In layer V of the motor cortex, iNOS-immunopositive neurons exhibit a staining localised in the whole soma as well as in the dendrites—the latter appearing particularly evident in male wildtype mice (Fig. 8D). Moreover, several iNOS-negative cells can be observed in the cortex of both *Brtl* groups (Fig. 8H and J). Quantitative analyses highlighted a significant reduction in iNOS-immunopositive CD in the cortex of *Brtl* mice of both sexes, yet more marked in male (F-WT vs F-*Brtl* *p* = 0.02; M-WT vs M-*Brtl* *p* < 0.001). Additionally, also a significant decrease of iNOS-immunopositive optical density was found in the cortical innermost pyramidal layer of both male and female *Brtl* mice (F-WT vs F-*Brtl* *p* = 0.006; M-WT vs M-*Brtl* *p* = 0.004) (Fig. 8; Tables S14 and S15).

iNOS immunohistochemical staining in the caudate-putamen revealed a scattered immunopositivity, primarily localised in the cytoplasm. While female groups did not show any major differences, in *Brtl* male mice iNOS staining appears fainter compared to male controls (Fig. 8). Consistently, quantitative analyses in the dorsal striatum revealed major sex-specific iNOS dysregulation: while no changes were detected in female animals, *Brtl* male showed a very significant reduction in immunopositive cell density (F-WT vs F-*Brtl* *p* = 0.27; M-WT vs M-*Brtl* *p* < 0.001) as well as in iNOS-OD of individual cells (F-WT vs F-*Brtl* *p* = 0.12; M-WT vs M-*Brtl* *p* = 0.009). Striosomal iNOS-OD showed no differences between mutant and wildtype groups(F-WT vs F-*Brtl* *p* = 0.12; M-WT vs M-*Brtl* *p* = 0.11) (Fig. 8; Tables S14 and S15).

### Transmission electron microscopy ultrastructural assessment in the motor cortex

As the histochemical and immunohistochemical analyses revealed marked changes in the brain of *Brtl* mice, transmission electron microscopy (TEM) was employed as a preliminary investigation to evaluate possible cortical changes at ultrastructural level.

**Fig. 9:**
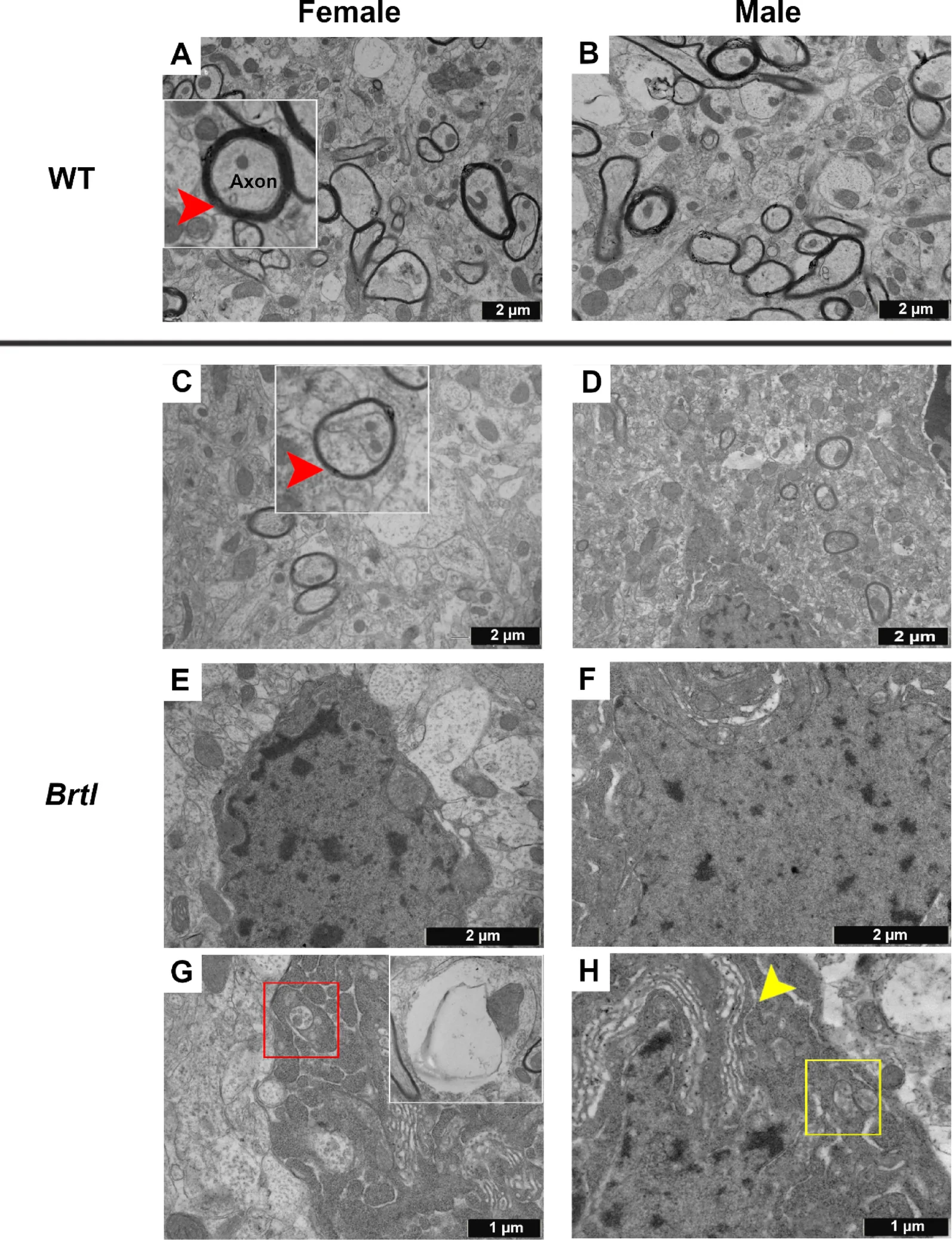
Representative micrographs of motor cortex specimens investigated by TEM, showing myelin distribution in wildtype (WT; **A** and **B**) and Brtl (**C** and **D**) mice, as well as abnormal morphology of oligodendrocytes in Brtl specimens (**E**–**H**). The red arrows in panel **A** and **C** exemplify myelin sheaths enveloping axons in wildtype and mutant mice, respectively. Panels **G** and **H** show higher magnifications of oligodendrocytes’ ultrastructural details from female and male Brtl specimens, respectively: the red box indicates multivescicular bodies, while the insert highlights the presence of autophagic events (panel **G**); the yellow arrowhead points to an irregular RER, while the yellow box highlights abnormal mitochondria, with redced cristae and less dense matrix (panel **H**). Magnification: 10 K × (**A**–**D**), 20 K × (**E** and **F**) and 30 K × (**G** and **H**)

**Fig. 10:**
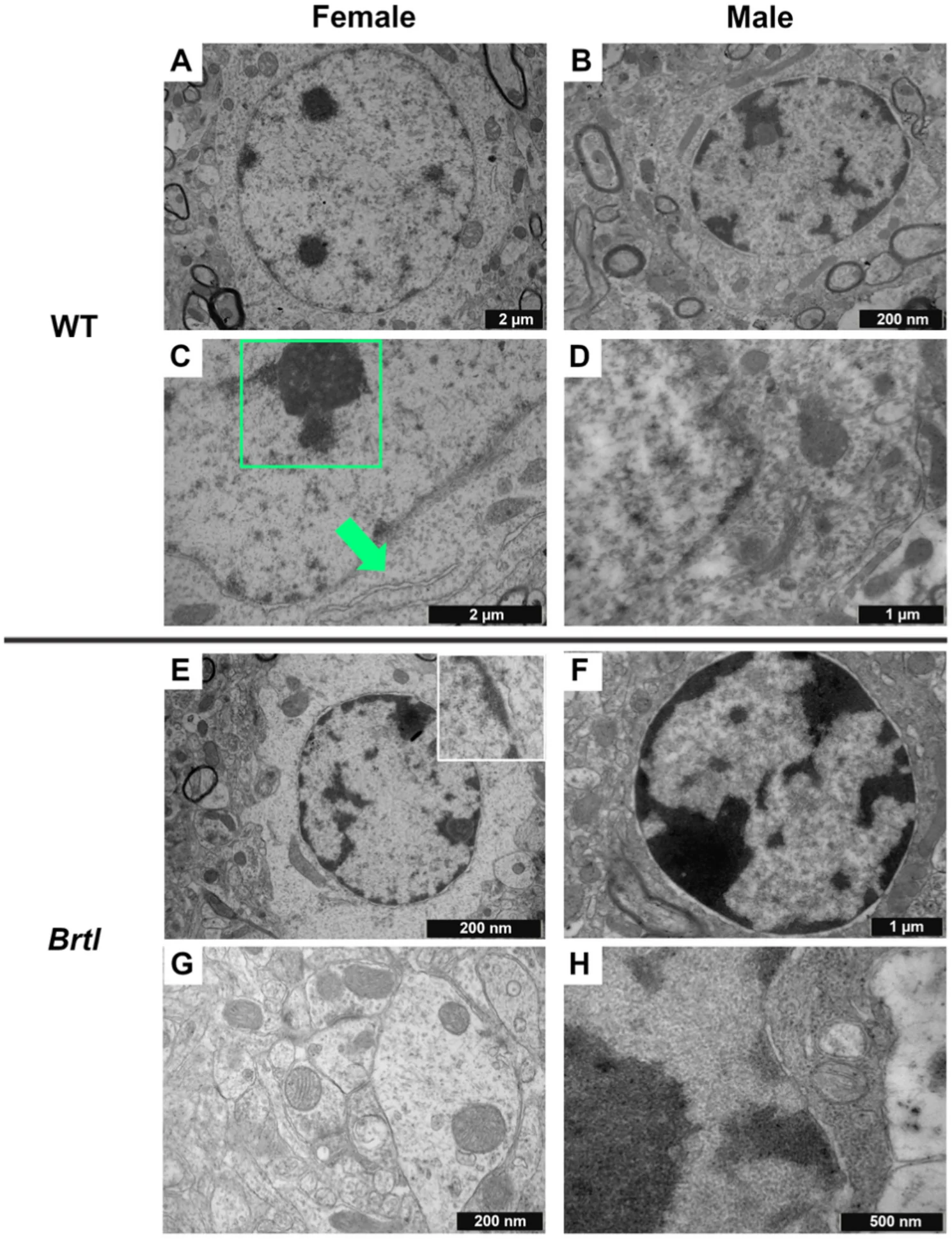
Representative micrographs of motor cortex specimens investigated by TEM, pyramidal neurons from wildtype (WT; **A**–**D**) and Brtl (**E**–**H**) mice. Panel **A** and **B** show pyramidal neurons from female and male control mice; panel **C** and **D** show higher magnifications of pyramidal neurons: the green box in panel **C** exemplifies the presence of numerous fibrillary centres in the nuclei of wildtype pyramidal neurons, while the green arrow indicates the well-defined and prominent RER; panel **D** shows prominent RER in male controls. Panel **E** and **F** exemplify pyramidal neurons from Brtl specimens, with less well-defined RER, rarefied cytoplasm and irregular nuclear envelope (panel **E**, insert) as well as abnormally condensed chromatin (panel **F**); panel **G** and **H** exemplify the frequent autophagic events detected in Brtl ultrathin sections. Magnification: 10 K × (**A** and **E**), 15 K × (**B**), 20 K × (**C**), 25 K × (**F**), 30 K × (**D** and **G**) and 75 K × (**H**)

TEM analysis in the innermost pyramidal layer of the motor cortex highlighted ultrastructural alterations both in neural and non-neuronal cell types of male and female *Brtl* mice (Figs. 9 and 10). Figure 9 shows TEM images of myelin distributrion and oligodendrocytes from ultrathin sections of motor cortex: subpanels A-B and C-H correspond to WT and Brtl mice, respectively; within each genotype, subpanels A and B-G represent females, whereas subpanels B and D–H represent males. TEM investigations evidenced reduced and, overall, more disorganised myelin in *Brtl* groups, (Fig. 9A–D). Consistently with this observation, we detected several oligodendrocytes with dysfunctional and stressed morphology in *Brtl* cortical specimens (Fig. 9E–H): some mitochondria showed reduced cristae and less densematrix (Fig. 9G and H), also multivesicular bodies and autophagic events were detectable (Fig. 9E). Oligodendrocytes in the motor cortex of mutant animals also display alterations in the rough endoplasmic reticulum (RER) that appears dilated and irregular compared to controls, particularly in male mice (Fig. 9H). These ultrastructural features are not found in oligodendrocytes under physiological conditions and are rather associated with pathological conditions [67, 68].

Cortical neurons’ ultrastructure can be recognised from their large nucleus, with a rim of heterochromatin lining the nuclear envelope, sparse euchromatin in the centre and a prominent nucleolus or, occasionally, nucleoli; moreover, a very vast RER (Nissl substance) and numerous mitochondria can be observed in the cytoplasm of large pyramidal neurons, supporting their high synthetic and metabolic activity [69]. Figure 10 shows TEM images of pyramidal neurons from ultrathin sections of motor cortex: subpanels A–D and E–H correspond to WT and Brtl mice, respectively; within each genotype, subpanels A, C, E, and G represent females, whereas subpanels B, D, F and H represent males. Our ultrastructural analysis of the motor cortex revealed the presence of shrunken neurons, more frequent in *Brtl* mice than in controls: these cells exhibit an altered and compacted morphology: rarefied cytoplasm with less evident RER, swallen and fragmented nuclear envelope and highly condensed chromatin, typical of pyknotic nuclei (Fig. 10E and F). Conversely, in control mice neurons show a well-preserved morphology: numerous nuclear fibrillary centres, indicators of intense protein synthesis, as well as prominent RER and intact mitochondria in the cytoplasm (Fig. 10A–D). *Brtl* mice also show a higher number of morphological features indicative of autophagy, particularly mitophagic events, compared to control animals, several of which are found within axons (Fig. 10G and H).

### Woelcke’s haematoxylin: myelin assessment in the cortex and striatum

In light of the evidence obtained from TEM analyses in the motor cortex, we further investigated potential alterations concerning myelin organisation and integrity, using Woelcke’s haematoxylin staining [60, 61].

**Fig. 11:**
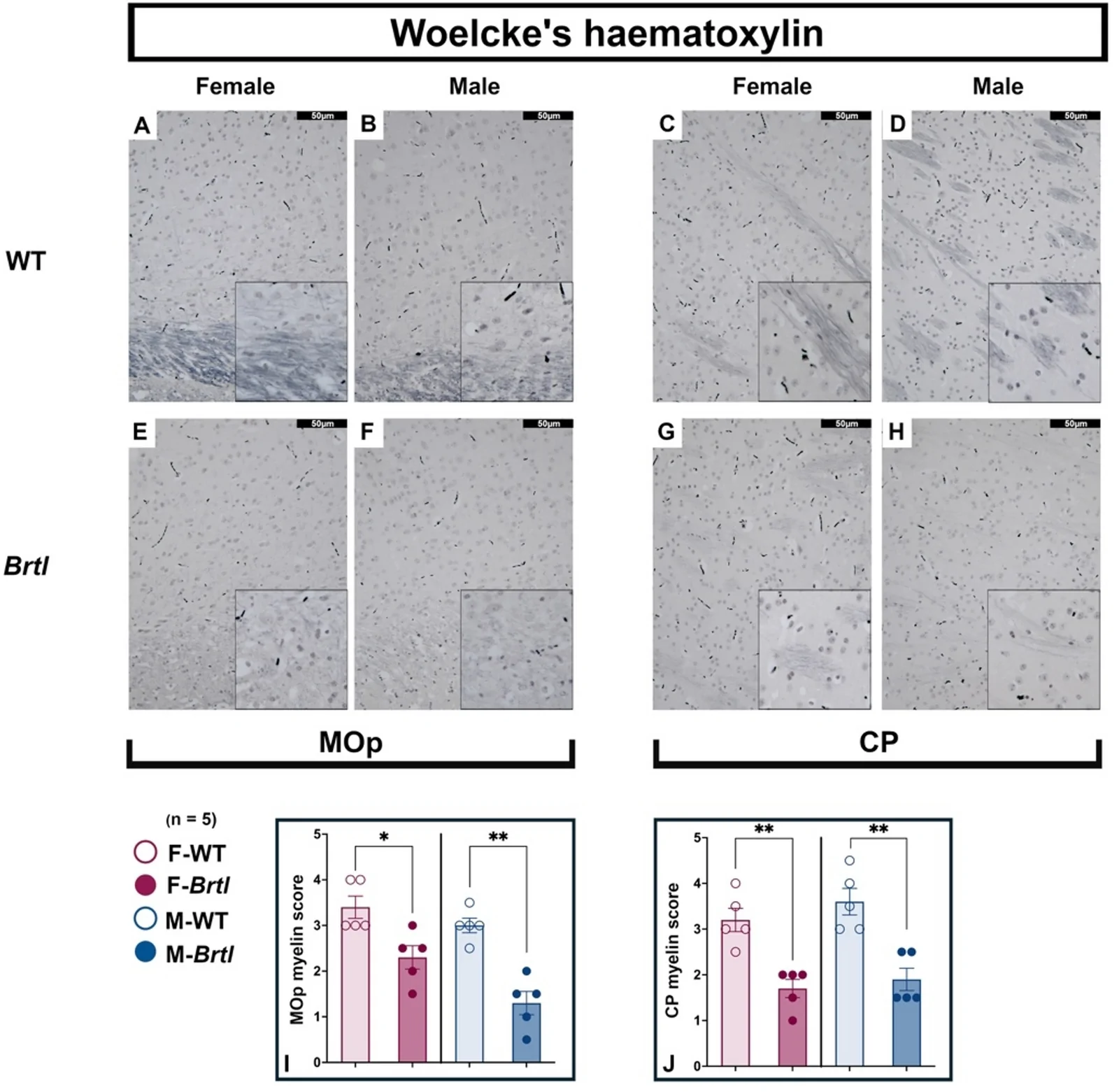
Representative micrographs and corresponding quantitative analyses of Woelcke’s haematoxylin histochemical staining in sagittal section of the primary motor cortex (MOp) and caudate-putamen (CP), from male (**B**, **D**, **F**, **H**) and female (**A**, **C**, **E**, **G**) wildtype (WT; **A**–**D**) and Brtl mice (**E**–**H**). Magnification: 20 × (inserts 40 ×). Bar graphs: myelin score in the corpus callosum/MOp layer VI (I) and in CP striosomes (J); *p*-values: (*) *p* < 0.05, and (**) *p* < 0.01

Figures 11 and S9 show representative images of myelin staining in the cortex, striatum and midbrain region using Woelcke’s haematoxylin. In Fig. 11, subpanels A–D and E–H correspond to WT and Brtl mice, respectively. Within each genotype, subpanels A, C, E, and G represent females, whereas subpanels B, D, F, and H represent males In control mice, an intense staining highlights dense and well-preserved myelinated fibers in the cortex innermost layers and corpus callosum, in the caudate-putamen striatal fibres (Fig. 11, inserts), and in the midbrain (Fig. S9). In contrast, mutant mice display markedly reduced staining intensity, suggesting a loss of myelin or defective myelination in *Brtl* mice of both sexes. Quantitative analyses indicated significant myelin defects in all three regions considered (Figs. 11 and S9; Table S16).

## Discussion

Although OI is classically considered a predominantly skeletal disorder, clinical reports have suggested possible involvement of the nervous system [14–17, 19]. However, the brain remains largely unexplored in this condition. In light of the locomotor deficits reported in the pathology –which prior works mainly attribute to alterd muscle structure and function, analyses were extended to motor-related brain regions [11–13, 70]; while previous studies have reported evidence of redox imbalances in *Brtl* cerebellar specimens, the marked regional heterogeneity of the brain in terms of cellular composition, metabolic activity and complementary functional roles of different motor areas, prompted the inclusion of functionally diverse regions beyond the cerebellum [71–74]. Accordingly, the motor cortex and caudate–putamen were investigated, given their roles in motor planning and execution, and in the integration and modulation of motor output, as opposed to the cerebellum which mainly participates incoordination and timing [75, 76]; in addition, a broader panel of oxidative stress markers and histological staining approaches was employed to further characterize redox alterations across these region. To our knowledge, the data reported in the present work provide a pioneering histological characterisation of the cortex and striatum in OI murine models, revealing alterations in the primary motor cortex and caudate-putamen of the Brtl mice, although functional implications (e.g., behavioral and electrophysiological analyses) still warrant further investigation. Further studies should also explore in greater detail the potential activation of specific cell death mechanisms (e.g. apoptosis) and their possible functional consequences in different OI brain areas, as well as potential morphological, ultrastructural, and molecular alterations in early adult animals (i.e., 12 or 26 weeks of age).

In accordance to previous findings regarding decreased cerebellar cell density in *Brtl* animals [37], the increased density of shrunken-hyperchromatic cortical neurons and the reduced striatal cell density could derive from an impaired CNS milieu, potentially caused by altered redox conditions and an impaired inflammatory environment, which may contribute to neuronal damage and, overtime, lead to neuronal loss [77]. Here we report a redox imbalance in the OI brain, with changes in the oxidative stress pathway in cortical and striatal regions involved in motor functions. Indeed**,** dysfunctional modifications affecting ECM constituents are closely linked to redox imbalance and oxidative damage, suggesting a reciprocal interplay that contributes to tissue impairment [78, 79]. Particularly, the pathophysiology of collagen-related disorders such as OI seems to involve mitochondrial dysfunctions, higher ROS generation and overall increased oxidative stress [35]. Consistently, the results collected in the present study highlighted widespread alterations in the oxidative stress pathway of *Brtl* mice CNS compared to wildtype animals. Immunohistochemical analyses revealed marked alterations in the expression levels of several oxidative stress markers (SOD1, SOD2, GPx4, COX2, COX IV and iNOS), which may suggest pronounced redox imbalance in the motor cortex and dorsal striatum of mutant animals.

### Redox imbalances

Superoxide dismutases (SODs) are a family of key antioxidant enzymes that protect cells from oxidative damage by catalysing the dismutation of the superoxide anion into molecular oxygen and hydrogen peroxide [80]. Cytoplasmic SOD1 and mitochondrial SOD2 are constitutively expressed in the physiological brain but can be upregulated during oxidative damage or inflammation [81–84]. The increased levels detected for SOD1 and SOD2 in the motor cortex and dorsal striatum of both male and female *Brtl* suggest a marked upregulation of SODs, which may represent a compensatory mechanism induced by a state of chronic oxidative stress. The differential upregulation between SOD1 and SOD2 displayed by striosomal patches may differentially confer vulnerability to oxidative damage in the caudate-putamen of mutant animals and may reflect the different cellular compartimentalization of the two enzymes: SOD1 is known to confer major protection against cytoplasmic ROS while SOD2 is primarily involved in mitochondrial ROS detoxification [85]; therefore, cells of the striatal matrix, may require higher levels of both SODs, while striosomal patches may rely more strongly on mitochondrial SOD.

GPx4, a selenoprotein antioxidant enzyme involved in the regulation of lipid peroxidation and ferroptosis, is essential for neuronal survival, particularly during oxidative stress, and shows region-specific patterns in the CNS [86–88]. In stricking contrast to SODs, *Brtl* mice displayed substantial downregulation of GPx4 levels in the motor cortex as well as in the caudate-putamen region. This finding suggests that while superoxide scavenging is upregulated, downstream or complementary antioxidant systems (those handling peroxides/lipid peroxidation) are compromised. Indeed, prolonged exposure to ROS leads to GSH depletion, which consequently results in feedback downregulation of GPx4 [89]. Yet, under conditions of chronic redox imbalance, cells may experience GSH depletion and GPx4 downregulation, ultimately leading to increased lipid peroxidation and ferroptosis; of note, transcriptomic studies highlighted a high susceptibility of striatal neurons to impairments in glutathione metabolism [90].

Likewise, COX-IV, subunit 4 of the cytochrome c oxidase complex in the mitochondrial respiratory chain, also appeared substantially reduced in cortical and striatal neurons of *Brtl* mice, indicating impaired mitochondrial functionality and increased redox imbalance. Indeed, COX-IV levels can be downregulated by excessive ROS and previous experimental studies have highlighted a substantial reduction of mitochondrial COX in OI mice muscles [33]. In turn, a reduction in COX-IV levels, induced by chronic oxidative stress, can result in mitochondrial impairments which reflects on the overall activity of highly metabolically active cells such as pyramidal and striatal neurons [91–94].

Distinctlively, COX2 and iNOS exhibited cell- and sex-specific patterns, respectively, suggesting differential compensatory mechanisms across neuronal and non-neuronal populations as well as between sexes. Responsible for the conversion of arachidonic acid into prostaglandin H2 (PGH2), which is then metabolized into PGE2, COX2 plays a dual role in the brain: while glial COX2 is primarily involved in neuroinflammatory processes, in glutamatergic pyramidal neurons COX2 constitutive expression regulates neurovascular coupling and contributes to overall neuronal homeostatis [95–97]. On the other hand, iNOS downregulation in *Brtl* specimens revealed a region- and sex-specific pattern: in the motor cortex both *Brtl* groups showed a marked reduction of iNOS levels, while in the caudate-putamen this effect was restricted to males, suggesting compensatory mechanisms influenced both by CNS location and sex. Indeed, iNOS is the inducible cytoplasmatic nitric oxide synthase, responsible for generating nitric oxide, a signaling molecule and free radical involved in neurotransmission and inflammation: chronic redox imbalances can trigger iNOS downregulation as an adaptive response: iNOS is tightly dependent on the presence of the cofactor tetrahydrobiopterin (BH_4_) that prevents the production of ROS in the place of NO synthesis; reduced BH_4_ levels can trigger iNOS downregulation [98]. iNOS expression is also influenced by hormonal factors: in females, estrogens can modulate BH_4_ regulation, contributing to its synthesis and thereby maintaining higher iNOS levels in *Brtl* females compared to male mutants and the impact may show differences between brain areas [99–101]. Overall, changes in COX2 and iNOS enzyme levels in Brtl specimens further support an imbalanced redox equilibrium and, from a speculative perspective, suggest that neuroinflammatory processes may also be involved, an aspect that should nonetheless be validated through the analysis of the expression levels of specific pro- and anti-inflammatory markers, as well as a careful direct analysis of ROS levels.

### Cortical ultrastructural changes

The ultrastructural analysis evidenced by electron microscopy highlighted substantial alterations in cortical neurons and glial cells of *Brtl* mice, particularly in pyramidal neurons and oligodendrocytes. Consistent with Nissl staining, we observed a higher number of shrunken degenerating neurons in mutant mice compared to the respective controls. Additionally, *Brtl* animals displayed mitochondrial and RER alterations, specifically in cells resembling oligodendrocytes’ morphology which is in line with previous findings regarding the cerebellum of *Brtl* mice [37]. The overall disrupted morphology displayed by *Brtl* oligodendtocytes are indicators of stress-related alterations, since abnormalities in mitochondrial cristae and RER are known to be bidirectionally linked to oxidative stress; consistelty, increased autophagic events in such cell types can represent a compensatory mechanism in response to intracellular stress [68, 102, 103]. In turn, when oligodendrocytes become dysfunctional, their ability to synthetise myelin is reduced, resulting in the reduction of myelination [77].

Taken together, the ultrastructural alterations detected in the motor cortex of *Brtl* mice suggest marked cellular stress as well as myelin abnormalities, which are imputable to a condition of chronic oxidative stress and align with our histochemical/immunohistochemical analyses.

### Myelin dysregulations

Produced by oligodendrocytes in the CNS, myelin sheaths not only contribute to the faster saltatory conduction of nerve impulses but also promote metabolic support and axonal integrity. Throughout the brain, myelination is thoroughly regulated through a complex crosstalk of cells and molecules, including ECM constituents, and redox imbalances and inflammation can compromise this process, leading to a reduction in myelin content [104–107]. The present work highlights potential myelin dysregulation and deficits in aged brains of *Brtl* animals; particularly, as hinted by TEM ultrastructural investigations and confirmed through histochemical analyses, mutated animals display substantial myelin reduction compared to controls. Since redox imbalances can compromise myelin organisation and integrity, our findings corroborate the hypothesis that *Brtl* mice may experience persistent oxidative stress in several CNS regions, including the motor cortex and striatum, as well as the midbrain region, which may ultimately lead to progressive loss of myelination in aged subjects, further supporting potential motor deficits in these animals, although behavioral validation remains to be assessed in future studies.

## Conclusions

In conclusion, this work reported morphological alterations in the motor cortex and caudate-putamen as well as dysfunctionalities in the oxidative stress pathway in *Brtl* brains. These findings suggest that chronic oxidative stress in presence of altered collagen, may lead to the loss of cortical and striatal integrity in mice affected by dominant OI, although further investigations are needed to clarify this causal-effect relationship and to evaluate potential functional implications. Further studies should also focus on better dissecting the contribution of neuroinflammatory events as well as on the possible involvement of the neurovascular component in OI.

## Supplementary Information

Below is the link to the electronic supplementary material.

Supplementary Material 1

## Data Availability

The data presented in this study are available in the article and in the supplementary materials.
