# Supplementary material for: Exploring the brain in osteogenesis imperfecta: insights from the motor cortex and striatum of the Brtl+/− murine model
Source: Acta Neuropathol Commun. 2026 Jul 30;14:223. doi: 10.1186/s40478-026-02393-w (PMC13712080; doi:10.1186/s40478-026-02393-w)
Supplement: Supplementary file 1 [file 40478_2026_2393_MOESM1_ESM.docx]

**Supplementary material**

**Fig. S1**: Schematic illustration of the semi-quantitative score (0-5) used to assess myelination levels in Woelcke’s haematoxylin-stained brain sections, here exemplified by the corpus callosum of the motor cortex; the blue lines represent myelin fibres: 0 corresponds to absent staining (i.e. no myelin fibres), 5 indicates a very strong staining (i.e. extremely dense myelination)

**Fig. S2**: Representative micrographs and corresponding quantitative analysed of COL1A1 immunohistochemical staining in sagittal section of the primary motor cortex (MOp), from male (B and E) and female (A and D) wildtype (WT; A and B) and Brtl mice (D and E). Magnification: 10× (inserts 40×). Bar graphs: COL1A1-immunopositive OD in meninges (C) and blood vessels (F). p-values: (**) p < 0.01, and (***) p < 0.001

**Fig. S3:** No-antibody control for SOD1 immunohistochemical staining. Magnification 20×.

**Fig. S4:** No-antibody control for SOD2 immunohistochemical staining. Magnification 20×.

**Fig. S5:** No-antibody control for GPx4 immunohistochemical reaction. Magnification 20×.

**Fig. S6:** No-antibody control for COX-IV immunohistochemical staining. Magnification 20×.

**Fig. S7:** No-antibody control for COX2 immunohistochemical staining. Magnification 20×.

**Fig. S8:** No-antibody control for iNOS immunohistochemical staining. Magnification 20×.

**Fig. S9:** Sagittal sections of midbrain area from wildtype (A and B) and Brtl (C and D) mice. Magnification: 20×. The histogram (a) shows the semi-quantitative evaluation of myelin content in the midbrain (E); p-values: (*) p < 0.05

**Table S1.** Statistical analysis for the quantitative evaluation of COL1A1 immunopositive OD in the meninges and blood vessels; (**) p < 0.05 and (***) p < 0.01. Data are reported as mean ± SEM

|  | Experimental group |  | Experimental group | Statistical test | *p*-value |
| --- | --- | --- | --- | --- | --- |
| Meninges | Female WT  (189.5 ± 1.287) | *vs* | Female *Brtl*  (157.9 ± 7.097) | t-test | ** |
|  | Male WT  (187.9 ± 1.109) | *vs* | Male *Brtl*  (158.4 ± 3.968) | t-test | *** |
| Blood vessels | Female WT  (173.1 ± 9.857) | *vs* | Female *Brtl*  (131.7 ± 6.462) | t-test | ** |
|  | Male WT  (177.9 ± 5.963) | *vs* | Male *Brtl*  (147.8 ± 6.643) | t-test | ** |

**Table S2.** Statistical analysis for the quantitative evaluation of shrunken cell density (n/mm^2^) in the motor cortex – pyramidal layer; (*) p < 0.05 and (**) p < 0.01. Data are reported as mean ± SEM

| Experimental group |  | Experimental group | Statistical test | *p*-value |
| --- | --- | --- | --- | --- |
| Female WT  (5.025 ± 1.250) | *vs* | Female *Brtl*  (18.45 ± 2.243) | t-test | ** |
| Male WT  (6.471±1.102) | *vs* | Male *Brtl*  (15.97± 2.729) | t-test | * |

**Table S3.** Statistical analysis for the quantitative evaluation of striatal cell density (n/mm^2^) in the caudate-putamen; (*) p < 0.05. Data are reported as mean ± SEM

| Experimental group |  | Experimental group | Statistical test | *p*-value |
| --- | --- | --- | --- | --- |
| Female WT  (1112 ± 81.85 n/mm^2^) | *vs* | Female *Brtl*  (843.2 ± 41.53 n/mm^2^) | t-test | * |
| Male WT  (1425 ± 92.60 n/mm^2^) | *vs* | Male *Brtl*  (1158 ± 61.33 n/mm^2^) | t-test | * |

**Table S4**. Statistical analysis for the quantitative evaluation of SOD1-immunopositive cell density (n/mm^2^) in the motor cortex and caudate-putamen; (*) p < 0.05, (**) p < 0.01 and (***) p < 0.001. Data are reported as mean ± SEM

|  | Experimental group |  | Experimental group | Statistical test | *p*-value |
| --- | --- | --- | --- | --- | --- |
| Motor cortex | Female WT  (71.21 ± 2.151 n/mm^2^) | *vs* | Female *Brtl*  (83.54 ± 3.610 n/mm^2^) | t-test | * |
|  | Male WT  (65.29 ± 2.936 n/mm^2^) | *vs* | Male *Brtl*  (87.18 ± 2.135 n/mm^2^) | t-test | *** |
| Caudate-putamen | Female WT  (320.1 ± 12.88 n/mm^2^) | *vs* | Female *Brtl*  (540.4 ± 43.74 n/mm^2^) | t-test | ** |
|  | Male WT  (612.7 ± 8.776 n/mm^2^) | *vs* | Male *Brtl*  (767.6 ± 40.95 n/mm^2^) | t-test | ** |

**Table S5**. Statistical analysis for the quantitative evaluation of SOD1-immunopositive cells OD and immunopositive striosomes OD in the motor cortex and caudate-putamen; (ns) non-significant, (*) p < 0.05 and (**) p < 0.01. Data are reported as mean ± SEM

|  | Experimental group |  | Experimental group | Statistical test | *p*-value |
| --- | --- | --- | --- | --- | --- |
| Motor cortex  immunopositive cells | Female WT  (159.1 ± 2.309) | *vs* | Female *Brtl*  (171.4 ± 2.818) | t-test | ** |
|  | Male WT  (157.3 ± 3.132) | *vs* | Male *Brtl*  (170.4 ± 1.685) | t-test | ** |
| Caudate-putamen  immunopositive cells | Female WT  (136.6 ± 2.011) | *vs* | Female *Brtl*  (146.0 ± 1.926) | t-test | ** |
|  | Male WT  (147.8 ± 2.910) | *vs* | Male *Brtl*  (156.8 ± 1.417) | t-test | * |
| Caudate-putamen  striosomes OD | Female WT  (88.89 ± 1.542) | *vs* | Female *Brtl*  (92.23 ± 1.034) | t-test | ns |
|  | Male WT  (87.69 ± 0.8118) | *vs* | Male *Brtl*  (89.46 ± 0.7052) | t-test | ns |

**Table S6**. Statistical analysis for the quantitative evaluation of SOD2-immunopositive cell density (n/mm^2^) in the motor cortex and caudate-putamen; (*) p < 0.05 and (***) p < 0.001

|  | Experimental group |  | Experimental group |  | Statistical test | *p*-value |
| --- | --- | --- | --- | --- | --- | --- |
| Motor cortex | Female WT  (51.85 ± 5.197 n/mm^2^) | *vs* | Female *Brtl*  (78.94 ± 6.516 n/mm^2^) |  | t-test | * |
|  | Male WT  (47.32 ± 3.367 n/mm^2^) | *vs* | Male *Brtl*  (66.55 ± 5.877 n/mm^2^) |  | t-test | * |
| Caudate-putamen | Female WT  (111.7 ± 11.13 n/mm^2^) | *vs* | Female *Brtl*  (220.8 ± 10.70 n/mm^2^) |  | t-test | *** |
|  | Male WT  (137.4 ± 7.534 n/mm^2^) | *vs* | Male *Brtl*  (222.5 ± 19.43 n/mm^2^) |  | t-test | *** |

**Table S7**. Statistical analysis for the quantitative evaluation of SOD2-immunopositive cells OD and immunopositive striosomes OD in the motor cortex and caudate-putamen; (**) p < 0.01 and (***) p < 0.001. Data are reported as mean ± SEM

|  | Experimental group |  | Experimental group | Statistical test | *p*-value |
| --- | --- | --- | --- | --- | --- |
| Motor cortex  immunopositive cells | Female WT  (164.8 ± 2.059) | *vs* | Female *Brtl*  (179.1 ± 1.914) | t-test | *** |
|  | Male WT  (147.4 ± 1.949) | *vs* | Male *Brtl*  (174.7 ± 2.878) | t-test | *** |
| Caudate-putamen  immunopositive cells | Female WT  (127.8 ± 0.6215) | *vs* | Female *Brtl*  (143.9 ± 1.884) | t-test | *** |
|  | Male WT  (128.4 ± 1.597) | *vs* | Male *Brtl*  (135.4 ± 0.5771) | t-test | ** |
| Caudate-putamen  striosomes OD | Female WT  (99.20 ± 1.672) | *vs* | Female *Brtl*  (112.3 ± 0.8351) | t-test | *** |
|  | Male WT  (106.6 ± 1.108) | *vs* | Male *Brtl*  (113.8 ± 1.768) | t-test | ** |

**Table S8.** Statistical analysis for the quantitative evaluation of GPx4-immunopositive cell density (n/mm^2^) in the motor cortex and caudate-putamen; (*) p < 0.05 and (**) p < 0.01. Data are reported as mean ± SEM

|  | Experimental group |  | Experimental group | Statistical test | *p*-value |
| --- | --- | --- | --- | --- | --- |
| Motor cortex | Female WT  (88.06 ± 3.317 n/mm^2^) | *vs* | Female *Brtl*  (67.93 ± 4.711 n/mm^2^) | t-test | ** |
|  | Male WT  (92.67 ± 3.704 n/mm^2^) | *vs* | Male *Brtl*  (74.05 ± 4.246 n/mm^2^) | t-test | * |
| Caudate-putamen | Female WT  (356.0 ± 13.06 n/mm^2^) | *vs* | Female *Brtl*  (275.7 ± 26.12 n/mm^2^) | t-test | * |
|  | Male WT  (656.2 ± 23.15 n/mm^2^) | *vs* | Male *Brtl*  (561.3 ± 29.80 n/mm^2^) | t-test | * |

**Table S9**. Statistical analysis for the quantitative evaluation of GPx4-immunopositive cells OD and immunopositive striosomes OD in the motor cortex and caudate-putamen; (*) p < 0.05, (**) p < 0.01 and (***) p < 0.001. Data are reported as mean ± SEM

|  | Experimental group |  | Experimental group | Statistical test | *p*-value |
| --- | --- | --- | --- | --- | --- |
| Motor cortex  immunopositive cells | Female WT  (171.5 ± 1.656) | *vs* | Female *Brtl*  (164.3 ± 0.7284) | t-test | ** |
|  | Male WT  (180.6 ± 1.320) | *vs* | Male *Brtl*  (157.0 ± 1.307) | t-test | *** |
| Caudate-putamen  immunopositive cells | Female WT  (138.4 ± 1.186) | *vs* | Female *Brtl*  (132.2 ± 1.959) | t-test | * |
|  | Male WT  (139.1 ± 1.272) | *vs* | Male *Brtl*  (133.3 ± 1.812) | t-test | * |
| Caudate-putamen  striosomes OD | Female WT  (91.21 ± 1.146) | *vs* | Female *Brtl*  (81.61 ± 0.2443) | t-test | *** |
|  | Male WT  (89.07 ± 1.548) | *vs* | Male *Brtl*  (81.33 ± 1.643) | t-test | ** |

**Table S10.** Statistical analysis for the quantitative evaluation of COXIV-immunopositive cell density (n/mm^2^) in the motor cortex and caudate-putamen; (ns) non-significant and (**) p < 0.01. Data are reported as mean ± SEM

|  | Experimental group |  | Experimental group | Statistical test | *p*-value |
| --- | --- | --- | --- | --- | --- |
| Motor cortex | Female WT  (60.20 ± 8.422 n/mm^2^) | *vs* | Female *Brtl*  (40.42 ± 2.097 n/mm^2^) | t-test | ns |
|  | Male WT  (74.86 ± 6.893 n/mm^2^) | *vs* | Male *Brtl*  (38.95 ± 2.234 n/mm^2^) | t-test | ** |
| Caudate-putamen | Female WT  (194.0 ± 23.50 n/mm^2^) | *vs* | Female *Brtl*  (86.61 ± 9.488 n/mm^2^) | t-test | ** |
|  | Male WT  (284.1 ± 21.64 n/mm^2^) | *vs* | Male *Brtl*  (152.4 ± 25.34 n/mm^2^) | t-test | ** |

**Table S11**. Statistical analysis for the quantitative evaluation of COXIV-immunopositive cells OD, immunopositive striosomes OD in the motor cortex and caudate-putamen; (*) p < 0.05, (**) p < 0.01 and (***) p < 0.001. Data are reported as mean ± SEM

|  | Experimental group |  | Experimental group | Statistical test | *p*-value |
| --- | --- | --- | --- | --- | --- |
| Motor cortex  immunopositive cells | Female WT  (181.9 ± 2.564) | *vs* | Female *Brtl*  (152.4 ± 1.035) | t-test | *** |
|  | Male WT  (182.3 ± 1.737) | *vs* | Male *Brtl*  (164.5 ± 1.386) | t-test | *** |
| Caudate-putamen  immunopositive cells | Female WT  (131.2 ± 0.8712) | *vs* | Female *Brtl*  (121.8 ± 0.9963) | t-test | *** |
|  | Male WT  (134.5 ± 1.731) | *vs* | Male *Brtl*  (130.5 ± 0.7532) | t-test | * |
| Caudate-putamen  striosomes OD | Female WT  (97.76 ± 0.9648) | *vs* | Female *Brtl*  (86.52 ± 1.323) | t-test | *** |
|  | Male WT  (93.08 ± 0.8699) | *vs* | Male *Brtl*  (89.57 ± 0.5092) | t-test | ** |

**Table S12.** Statistical analysis for the quantitative evaluation of COX2-immunopositive cell density (n/mm^2^) in the motor cortex and caudate-putamen; (*) p < 0.05, (**) p < 0.01 and (***) p < 0.001. Data are reported as mean ± SEM

|  | Experimental group |  | Experimental group | Statistical test | *p*-value |
| --- | --- | --- | --- | --- | --- |
| Motor cortex | Female WT  (71.11 ± 6.360 n/mm^2^) | *vs* | Female *Brtl*  (53.58 ± 1.646 n/mm^2^) | t-test | * |
|  | Male WT  (77.25 ± 5.924 n/mm^2^) | *vs* | Male *Brtl*  (51.58 ± 6.603 n/mm^2^) | t-test | * |
| Caudate-putamen | Female WT  (214.4 ± 16.30 n/mm^2^) | *vs* | Female *Brtl*  (276.7 ± 7.292 n/mm^2^) | t-test | ** |
|  | Male WT  (233.9 ± 16.30 n/mm^2^) | *vs* | Male *Brtl*  (389.8 ± 25.41 n/mm^2^) | t-test | *** |

**Table S13**. Statistical analysis for the quantitative evaluation of COX2-immunopositive cells OD and immunopositive striosomes OD in the motor cortex and caudate-putamen; (ns) non-significant, (*) p < 0.05 and (***) p < 0.001. Data are reported as mean ± SEM

|  | Experimental group |  | Experimental group | Statistical test | *p*-value |
| --- | --- | --- | --- | --- | --- |
| Motor cortex  immunopositive cells | Female WT  (167.1 ± 1.844) | *vs* | Female *Brtl*  (143.6 ± 3.486) | t-test | *** |
|  | Male WT  (181.8 ± 1.867) | *vs* | Male *Brtl*  (159.4 ± 3.342) | t-test | *** |
| Caudate-putamen  immunopositive cells | Female WT  (146.7 ± 1.867) | *vs* | Female *Brtl*  (154.7 ± 2.417) | t-test | * |
|  | Male WT  (147.7 ± 1.807) | *vs* | Male *Brtl*  (154.8 ± 2.076) | t-test | * |
| Caudate-putamen  striosomes OD | Female WT  (84.86 ± 0.3254) | *vs* | Female *Brtl*  (84.14 ± 0.4371) | t-test | ns |
|  | Male WT  (84.66 ± 0.2827) | *vs* | Male *Brtl*  (85.65 ± 0.4618) | t-test | ns |

**Table S14.** Statistical analysis for the quantitative evaluation of iNOS-immunopositive cell density (n/mm^2^) in the motor cortex and caudate-putamen; (ns) non-significant, (*) p < 0.5 and (***) p < 0.001. Data are reported as mean ± SEM

|  | Experimental group |  | Experimental group | Statistical test | *p*-value |
| --- | --- | --- | --- | --- | --- |
| Motor-cortex | Female WT  (52.22 ± 5.688 n/mm^2^) | *vs* | Female *Brtl*  (30.42 ± 4.353 n/mm^2^) | t-test | * |
|  | Male WT  (54.90 ± 3.424 n/mm^2^) | *vs* | Male *Brtl*  (26.50 ± 2.605 n/mm^2^) | t-test | *** |
| Caudate-putamen | Female WT  (85.04 ± 2.360 n/mm^2^) | *vs* | Female *Brtl*  (84.59 ± 2.432 n/mm^2^) | t-test | ns |
|  | Male WT  (78.46 ± 6.867 n/mm^2^) | *vs* | Male *Brtl*  (40.03 ± 2.416 n/mm^2^) | t-test | *** |

**Table S15**. Statistical analysis for the quantitative evaluation of iNOS-immunopositive cells OD and immunopositive striosomes OD in the motor cortex and caudate-putamen; (ns) non-significant and (**) p < 0.01. Data are reported as mean ± SEM

|  | Experimental group |  | Experimental group | Statistical test | *p*-value |
| --- | --- | --- | --- | --- | --- |
| Motor cortex  immunopositive cells | Female WT  (158.6 ± 3.455) | *vs* | Female *Brtl*  (143.8 ± 1.876) | t-test | ** |
|  | Male WT  (153.5 ± 2.299) | *vs* | Male *Brtl*  (143.3 ± 1.004) | t-test | ** |
| Caudate-putamen  immunopositive cells | Female WT  (125.7 ± 0.5780) | *vs* | Female *Brtl*  (123.8 ± 0.9162) | t-test | ns |
|  | Male WT  (128.9 ± 1.908) | *vs* | Male *Brtl*  (120.5 ± 1.521) | t-test | ** |
| Caudate-putamen  striosomes OD | Female WT  (80.14 ± 0.7123) | *vs* | Female *Brtl*  (81.78 ± 0.6166) | t-test | ns |
|  | Male WT  (83.51 ± 0.7055) | *vs* | Male *Brtl*  (81.11 ± 1.144) | t-test | ns |

**Table S16**. Statistical analysis for the semi-quantitative evaluation of the myelin score in the motor cortex, caudate-putamen and midbrain; (*) p < 0.05 and (**) p < 0.01. Data are reported as mean ± SEM

|  | Experimental group |  | Experimental group | Statistical test | *p*-value |
| --- | --- | --- | --- | --- | --- |
| Motor cortex | Female WT  (3.400 ± 0.2667) | *vs* | Female *Brtl*  (2.300 ± 0.2603) | Mann-Whitney | * |
|  | Male WT  (3.000 ± 0.2108) | *vs* | Male *Brtl*  (1.300 ± 0.3350) | Mann-Whitney | ** |
| Caudate-putamen | Female WT  (3.200 ± 0.2494) | *vs* | Female *Brtl*  (1.700 ± 0.2603) | Mann-Whitney | ** |
|  | Male WT  (3.600 ± 0.3055) | *vs* | Male *Brtl*  (1.900 ± 0.2333) | Mann-Whitney | ** |
| Midbrain | Female WT  (3.600 ± 0.2449) | *vs* | Female *Brtl*  (2.400 ± 0.2449) | Mann-Whitney | * |
|  | Male WT  (4.200 ± 0.3742) | *vs* | Male *Brtl*  (2.000 ± 0.3162) | Mann-Whitney | * |
